# Supplementary material for: A single-cell nanocoating of probiotics for enhanced amelioration of antibiotic-associated diarrhea
Source: Nat Commun. 2022 Apr 19;13:2117. doi: 10.1038/s41467-022-29672-z (PMC9019008; doi:10.1038/s41467-022-29672-z)
Supplement: Supplementary file 1 — Supplementary Information [file 41467_2022_29672_MOESM1_ESM.doc]

**Supplementary Information: A single-cell nanocoating of probiotics for enhanced amelioration of antibiotic-associated diarrhea**

Jiezhou Pan# , Guidong Gong# , Qin Wang, Jiaojiao Shang, Yunxiang He, Chelsea Catania, Dan Birnbaum, Yifei Li, Zhijun Jia, Yaoyao Zhang*, Neel S. Joshi*, Junling Guo*

# J.P. and G.G. contributed equally to this work.

*Corresponding authors

**Table S1.** The MBC of six different antibiotics to three probiotic samples.

|  | Ciprofloxacin  μg/mL | Tobramycin  μg/mL | Neomycin  μg/mL | Levofloxacin  μg/mL | Norfloxacin  μg/mL | Gentamycin  μg/mL |
| --- | --- | --- | --- | --- | --- | --- |
| *E. coli* Nissle1917 | 1.5625 | 25 | 25 | 3.125 | 1.5625 | 100 |
| *L. casei* ATCC393T | 300 | 50 | 25 | 25 | 300 | 100 |
| CVS HPC | 100 | 50 | 50 | 12.5 | 200 | 50 |

**Table S2. The types of probiotic strains** in commercial blends of CVS HPC.

| Probiotic | Amount per Serving % Daily Value |
| --- | --- |
| *Bifidobacterium bifidum* | ** |
| *Bifidobacterium breve* | ** |
| *Bifidobacterium longum* | ** |
| *Lactobacillus acidophilus* | ** |
| *Lactobacillus casei* | ** |
| *Lactobacillus helveticus* | ** |
| *Lactobacillus rhamnosus* | ** |
| *Lactobacillus plantarum* | ** |
| *Lactobacillus lactis* | ** |
| *Streptococcus thermophilus* | ** |

** Daily Value not established

**Table S3. Primers for RT-qPCR**

| **Primers** | **Primer sequence** | |
| --- | --- | --- |
|  | Forward (5′-3′) | Reverse (5′-3′) |
| **IL-10** | GGACAACATACTGCTAACCGACTC | AAAATCACTCTTCACCTGCTCCAC |
| **IL-6** | GTTCTCTGGGAAATCGTGGA | TGTACTCCAGGTAGCTA |
| **IL-1β** | TCCATGAGCTTTGTACAAGGA | AGCCCATACTTTAGGAAGACA |
| **TNF-α** | AGACCCTCACACTCAGATCA | TCTTTGAGATCCATGCCGTTG |
| **Occludin** | ATGTCCGGCCGATGCTCTC | TTTGGCTGCTCTTGGGTCTGTA |
| **Claudin-1** | CGGGCAGATACAGTGCAAAG | ACTTCATGCCAATGGTGGAC |

**
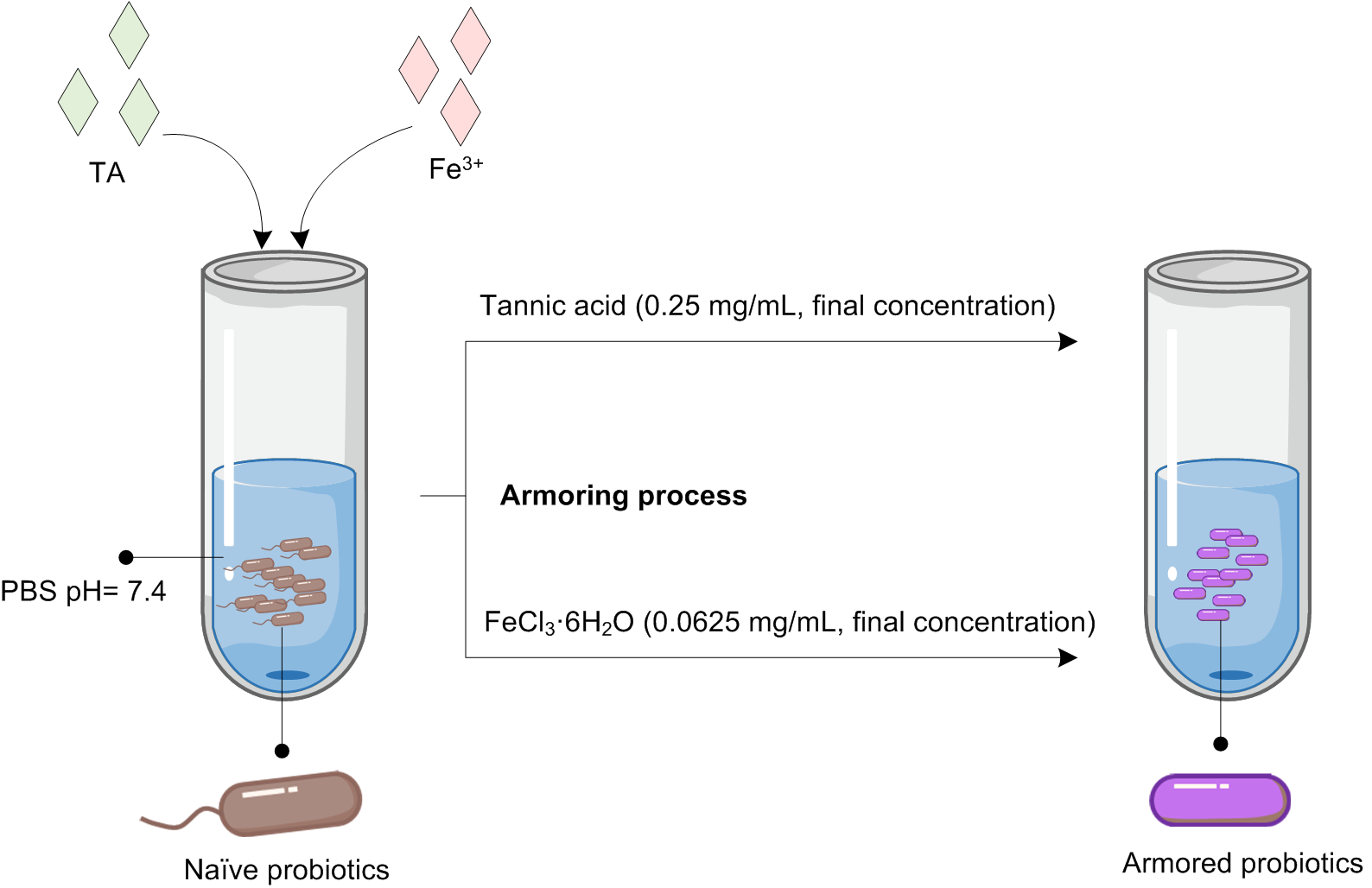
**

**Figure S1.** Schematic representation of the nanoarmor onto probiotics assembly method.


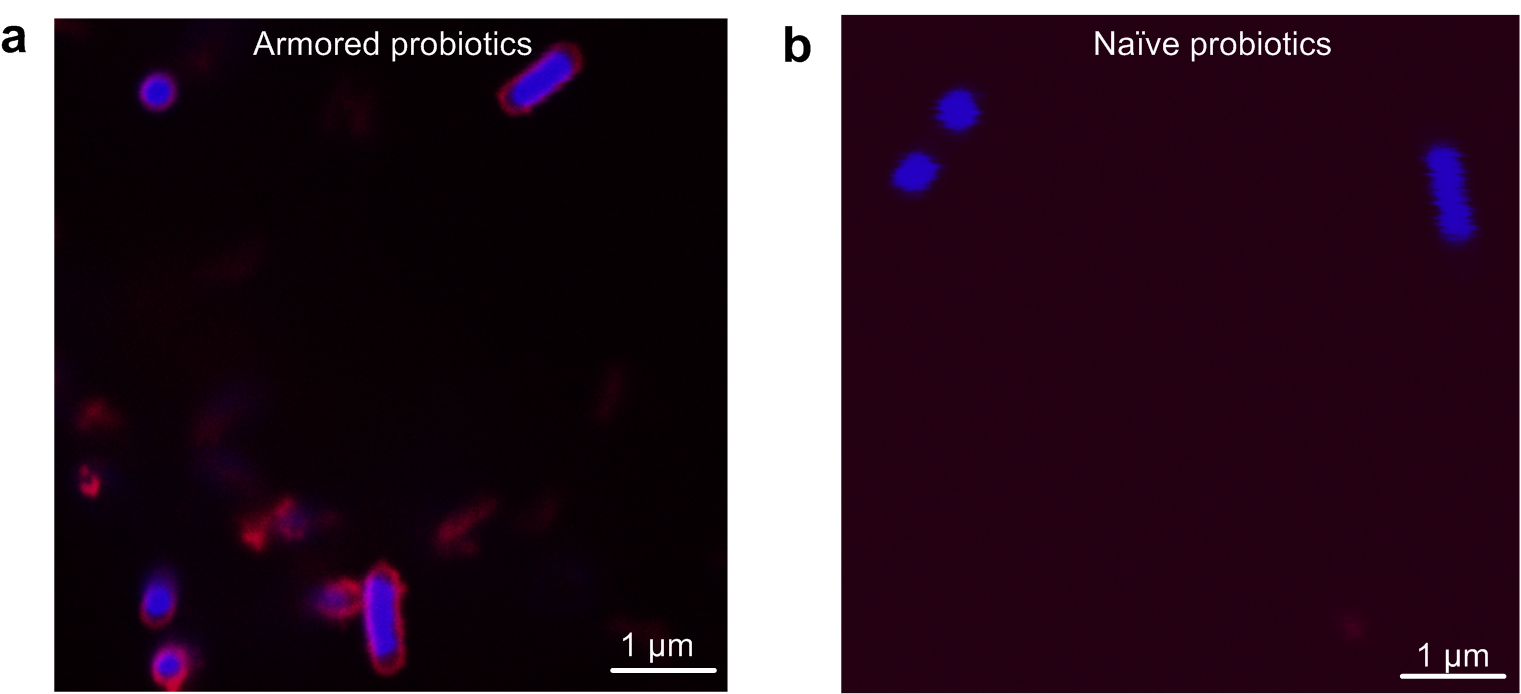


**Figure S2.** CLSM images of (**a**) armored and (**b**) naïve EcN. Nanoarmor was labeled with bovine serum albumin conjugated with Alexa Fluor 647. EcN was labeled with Hoechst. The results showed that BSA-Alexa-647 could not label naïve probiotics.

**
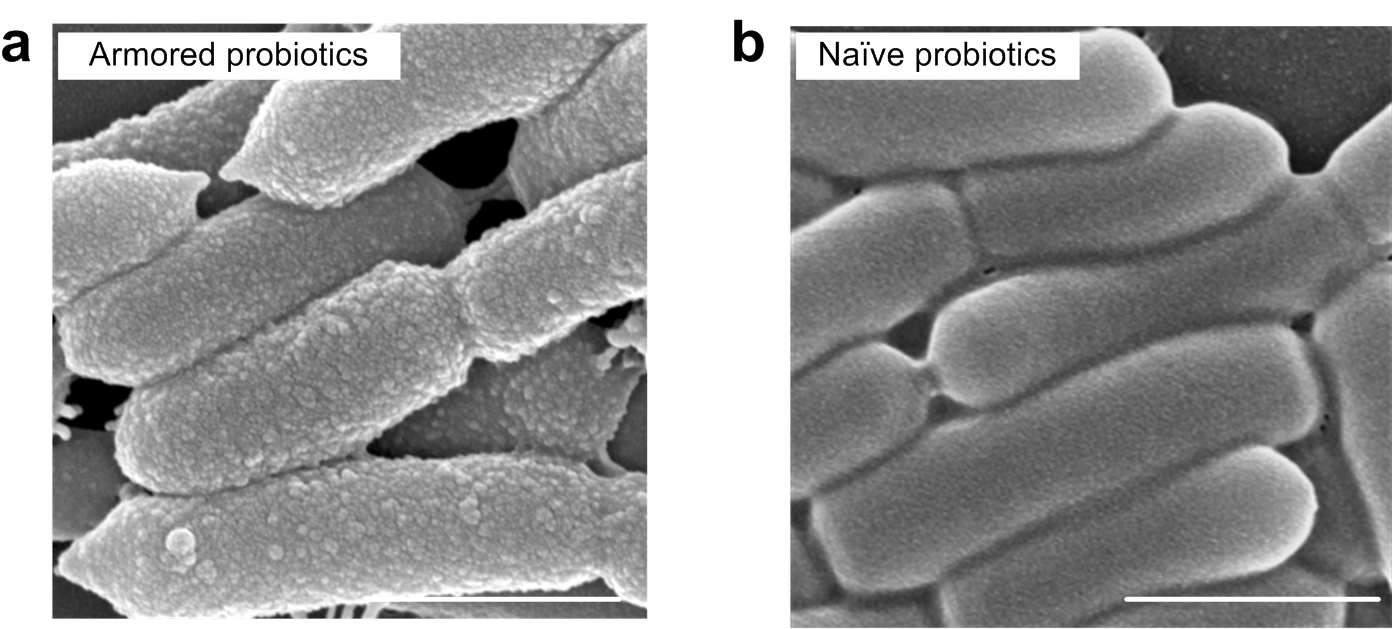
**

**Figure S3.** SEM images of (**a**) armored and (**b**) naïve EcN. Scale bars, 500 nm. The result showed that the nanoarmor was uniform.


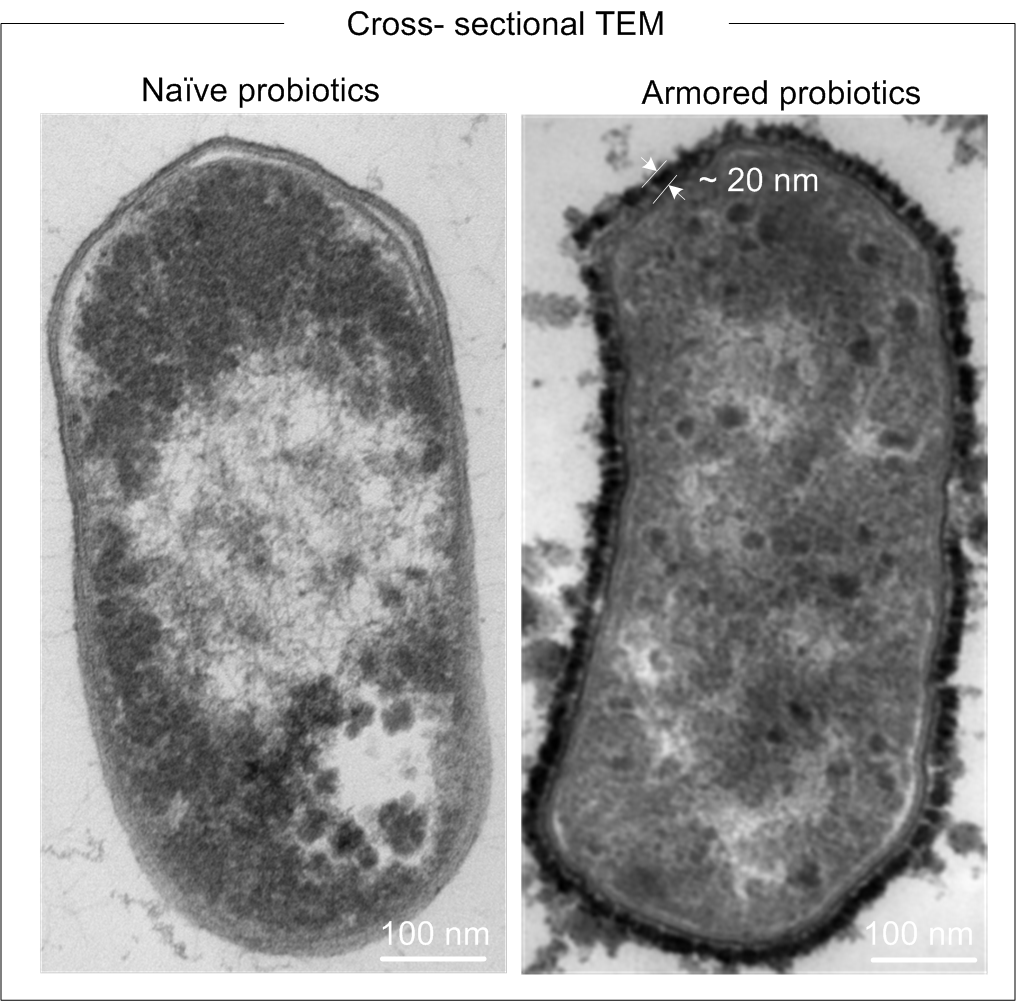


**Figure S4.** Cross-sectional TEM images of naïve or armored EcN. Scale bars, 100 nm. The result showed that the nanoarmor was uniform and ~20 nm thick.


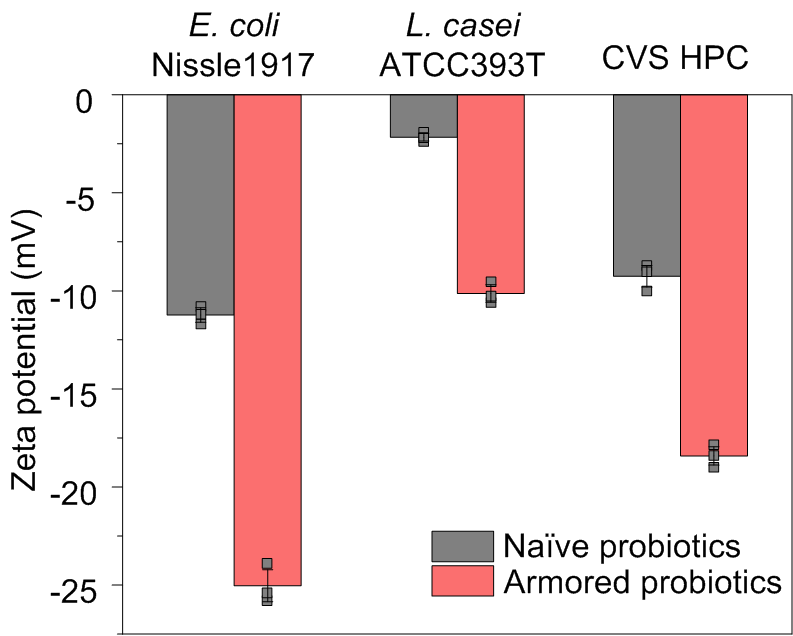


**Figure S5.** Zeta potential of naïve or armored probiotics. The variation is represented by the standard deviation of three independent replicates in all graphs. The graphs represent mean values ± SEM.


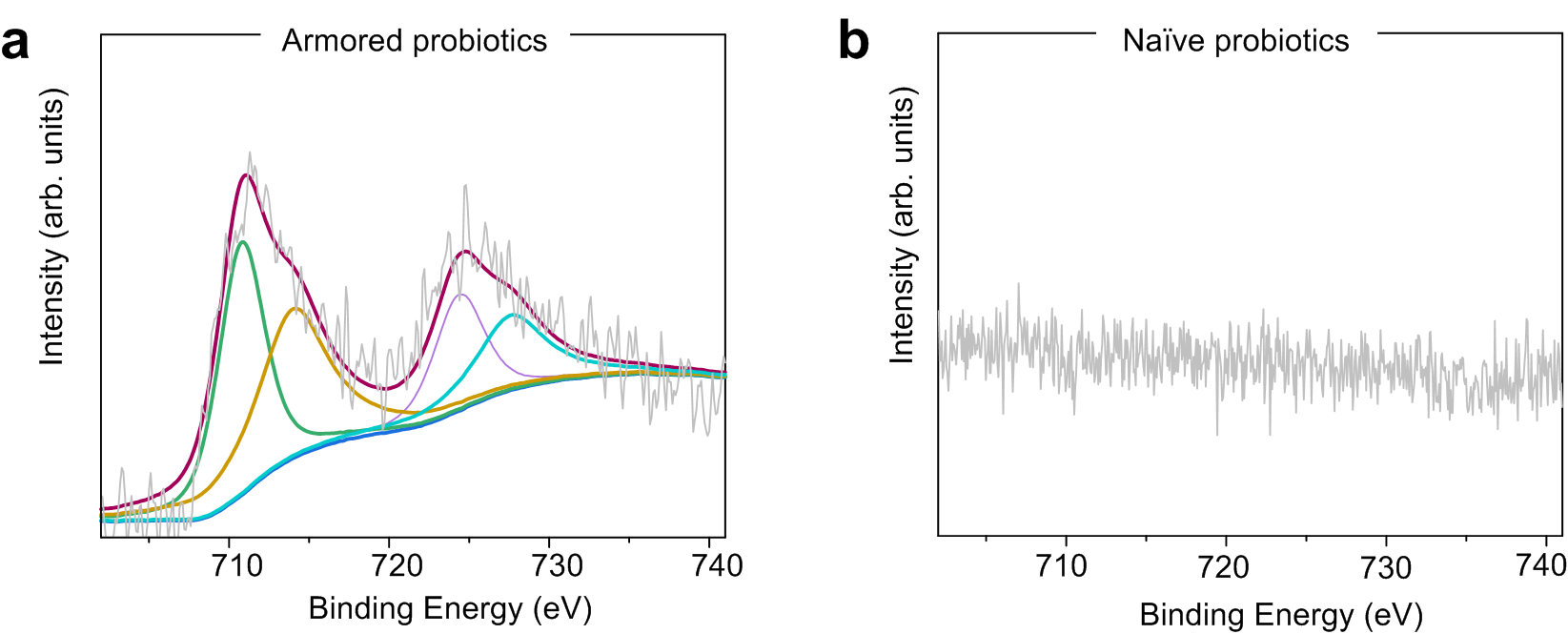


**Figure S6.** XPS spectrum of (**a**) armored or (**b**) naïve probiotics probiotic. The results showed the presence of Fe^III^ in armored probiotics.


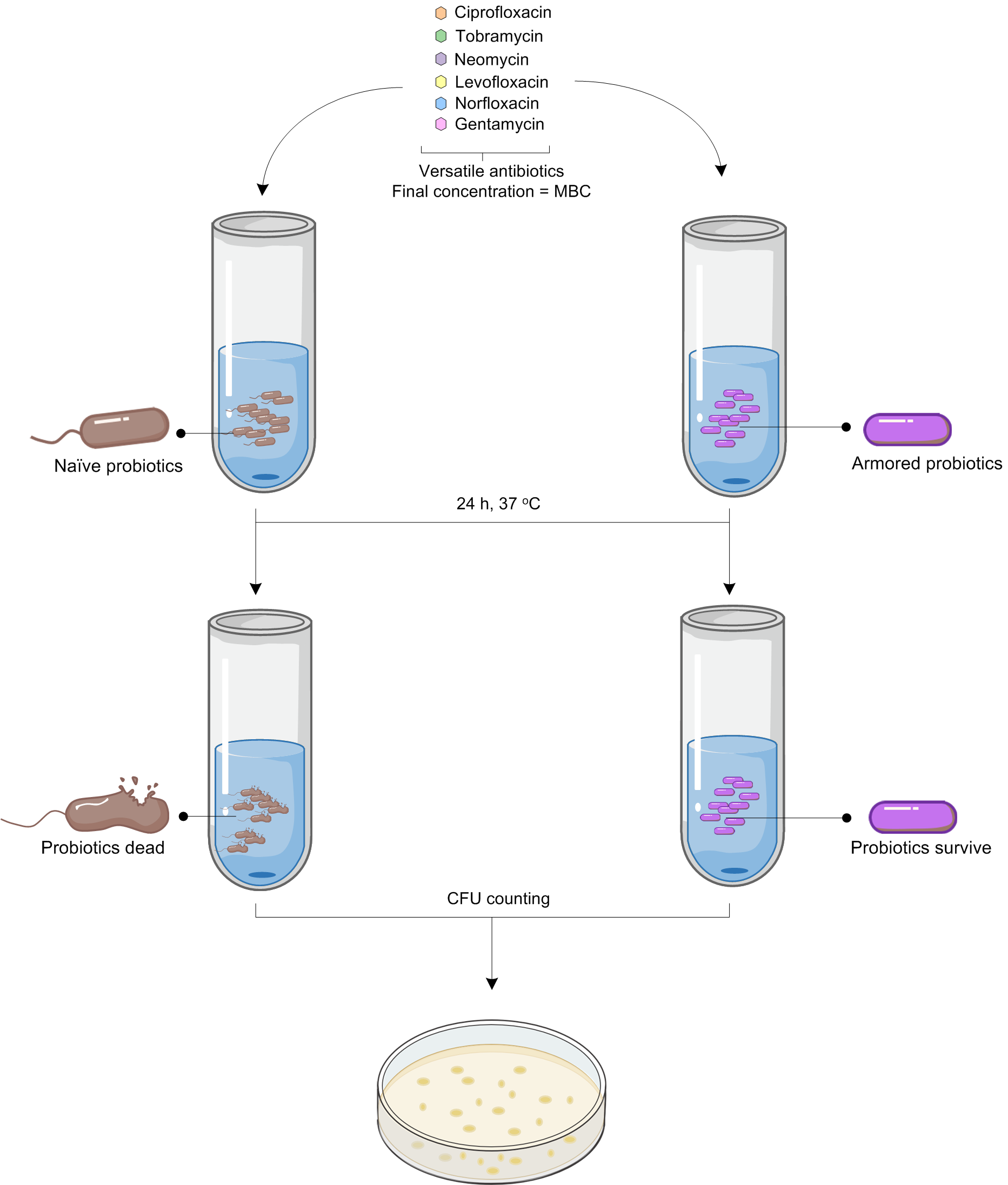


**Figure S7.** Schematic representation of the experiment for the armored probiotics against antibiotics.


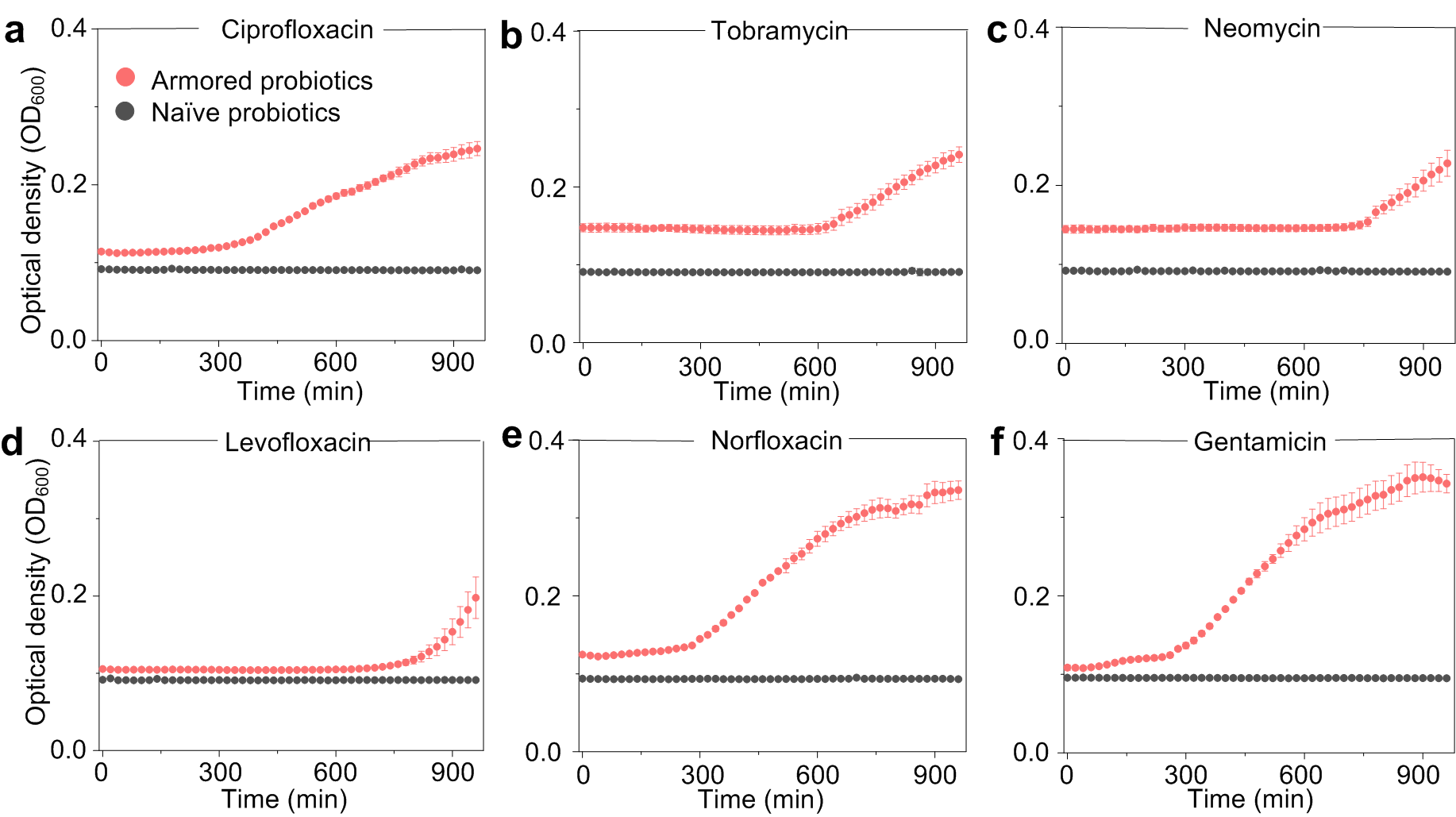


**Figure S8.** Growth curve of *E. coli* Nissle1917 and armored *E. coli* Nissle1917 in LB media after treating with (**a**) ciprofloxacin, (**b**) tobramycin, (**c**) neomycin, (**d**) levofloxacin, (**e**) norfloxacin, and (**f**) gentamicin for 24 hours. The variation is represented by the standard deviation of three independent replicates in all graphs. The graphs represent mean values ± SEM.


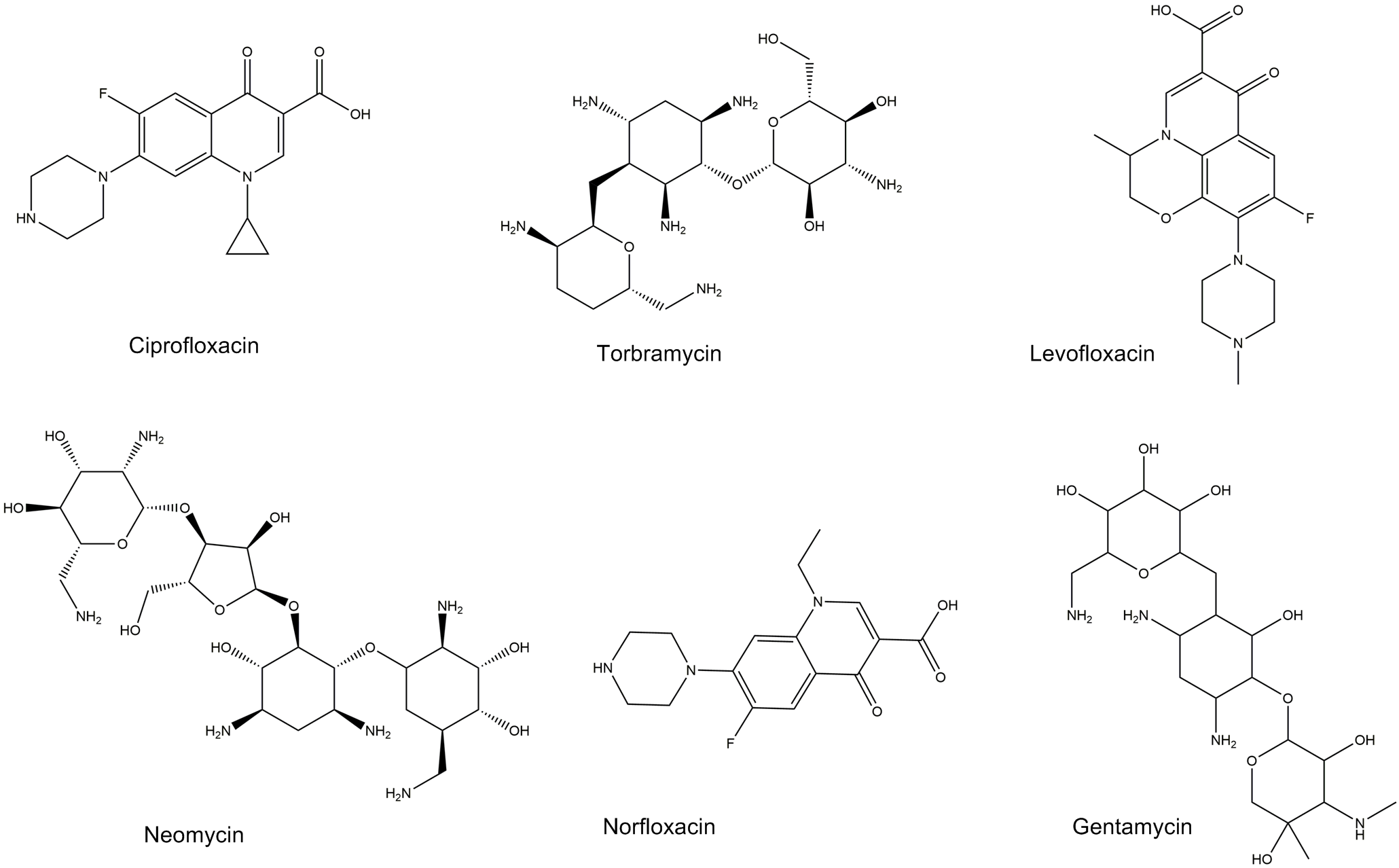


**Figure S9.** The molecular structures of six antibiotics used in this assay. These antibiotics have different molecular structures and mechanisms of action, suggested that the protective mechanism of the nanoarmor is generalizable.


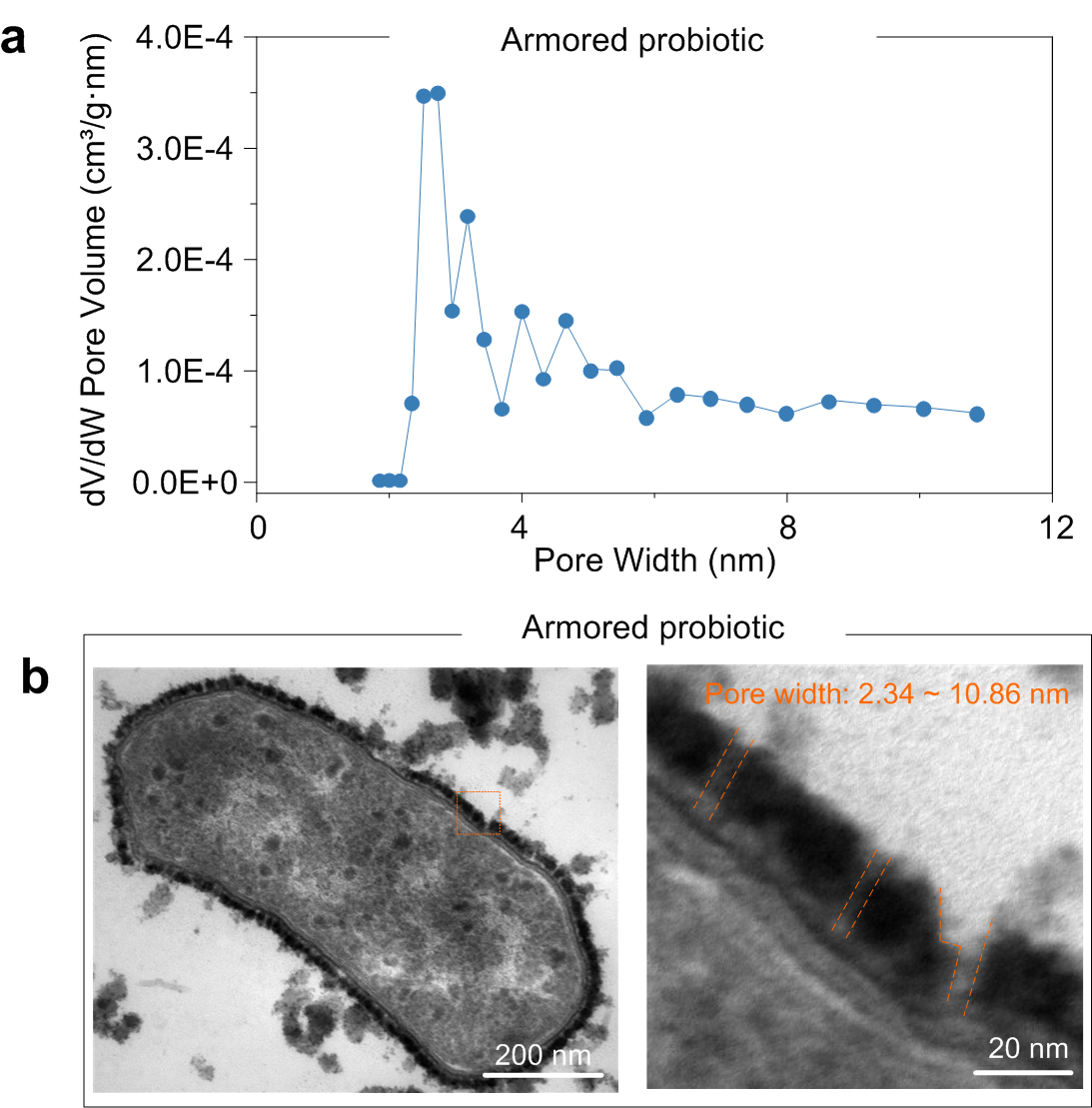


**Figure S10.** (**a**) BET result of armored probiotics, which showed a typical microporous structure with a pore diameter range from 2.34 - 10.86 nm. (**b**) The cross-sectional TEM showed that the nanoarmor was constructed by the assembled nanoparticles which densely distributed on the cell surface with gaps of up to 10 nm.


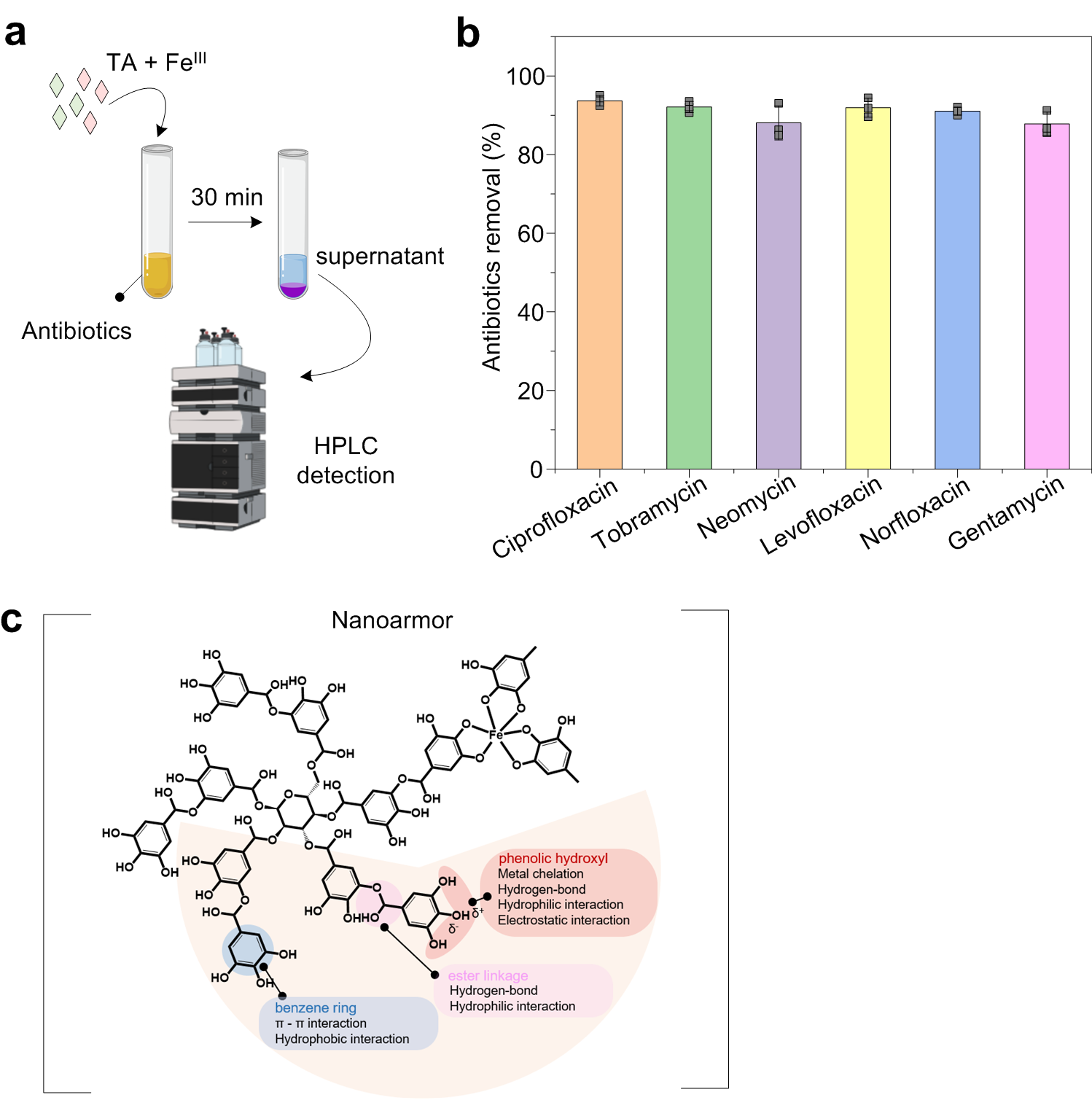


**Figure S11.** (**a**) Scheme of assays for the antibiotic adsorption ability of nanoarmor. (**b**) The adsorption effect of nanoarmor on six antibiotics. (**c**) The nanoarmor owns the ability to absorb antibiotics by the multiple interactions between the TA and antibiotics. Nanoarmor can absorb more than 90% of antibiotics and create a long-term microenvironment with low antibiotics concentration around the probiotic cells. Variation is represented by the standard deviation of three independent replicates in all graphs. The graphs represent mean values ± SEM.


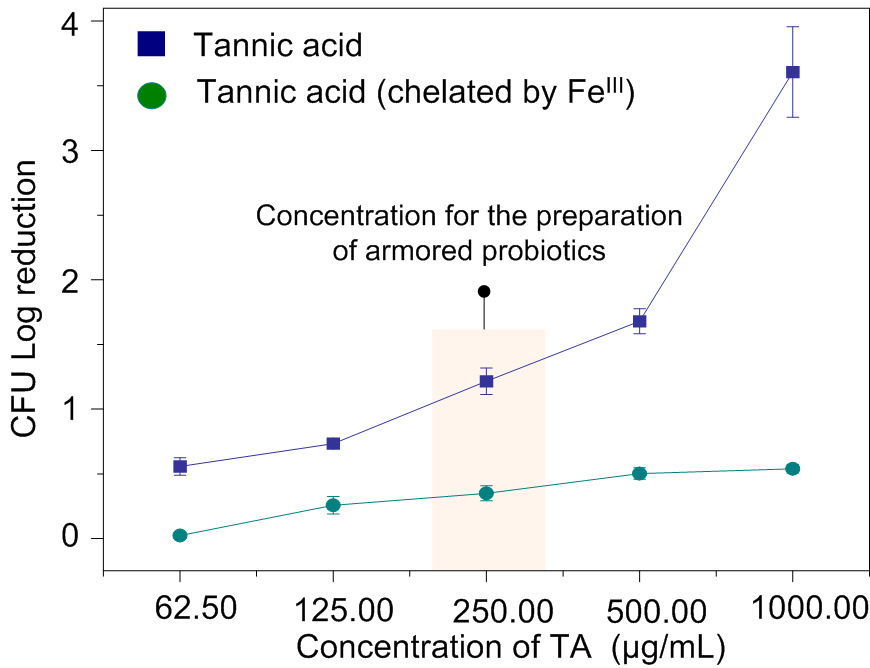


**Figure S12.** Log reduction of the TA/Fe^III^-TA complexes against EcN calculated from the CFU counting. The chelation of iron ions with TA reduces the antibacterial ability of TA. Variation is represented by the standard deviation of three independent replicates in all graphs. The graphs represent mean values ± SEM.


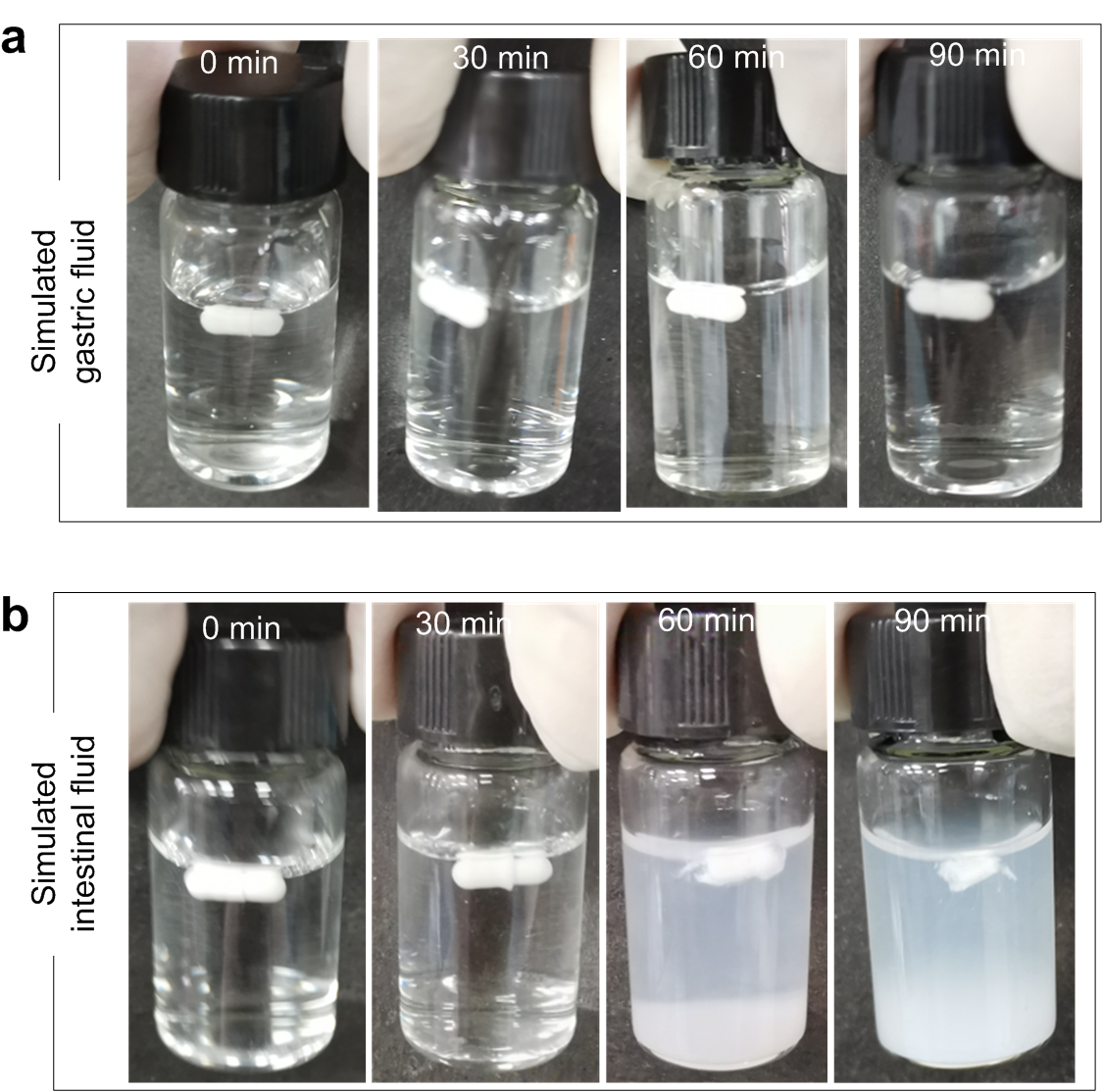


**Figure S13.** The photographs of the enteric capsules after treatment in (**a**) simulated gastric fluid and (**b**) simulated intestinal fluid. The enteric capsules did not dissolve in simulated gastric fluid and disintegrated rapidly in simulated intestinal fluid.


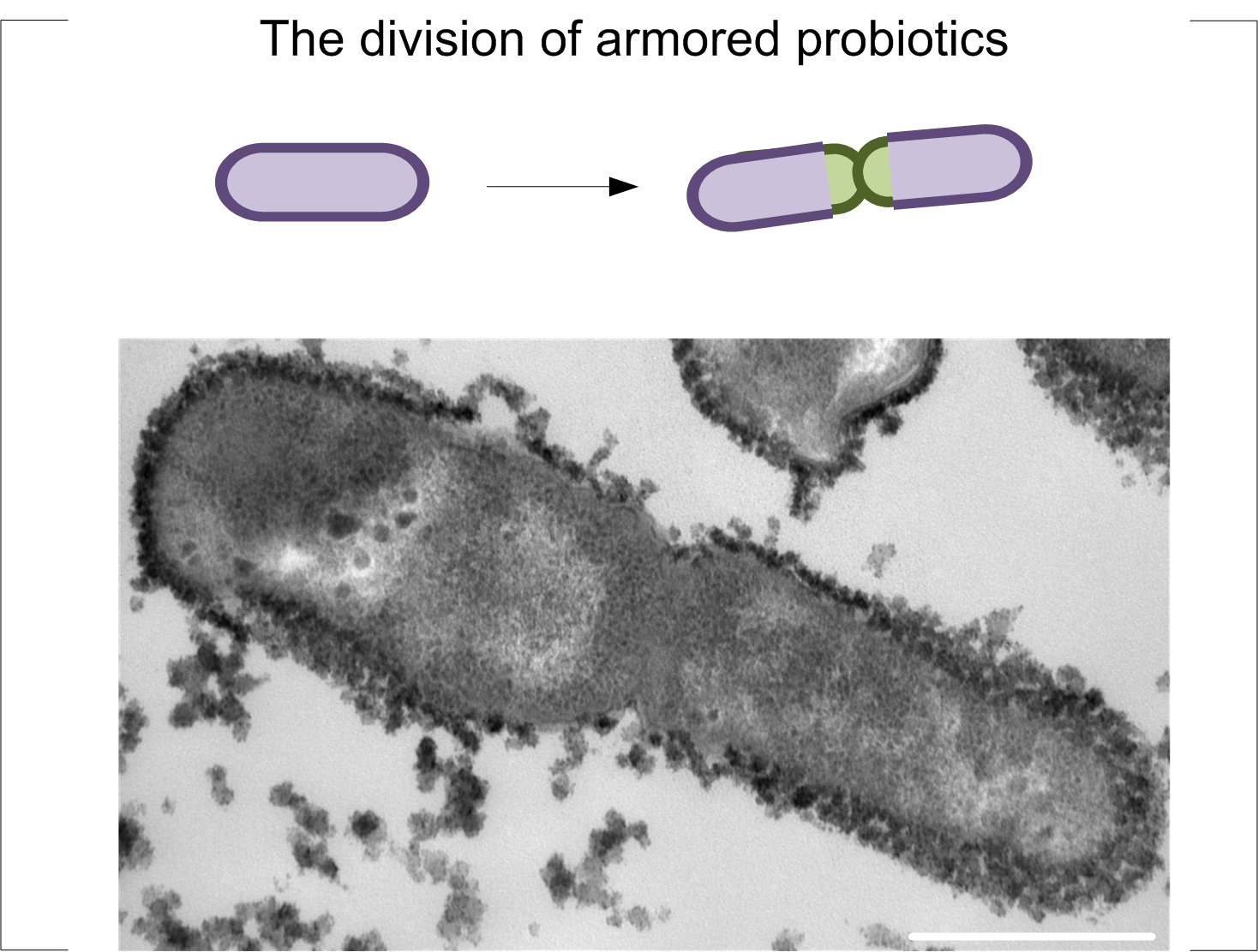


**Figure S14.** The cross-sectional TEM image of EcN dividing and breaking through the shell of nanoarmor. The nanoarmor could not affect the growth of the bacteria in media, which may be because of the nanoarmor shedding off after the division of bacteria.


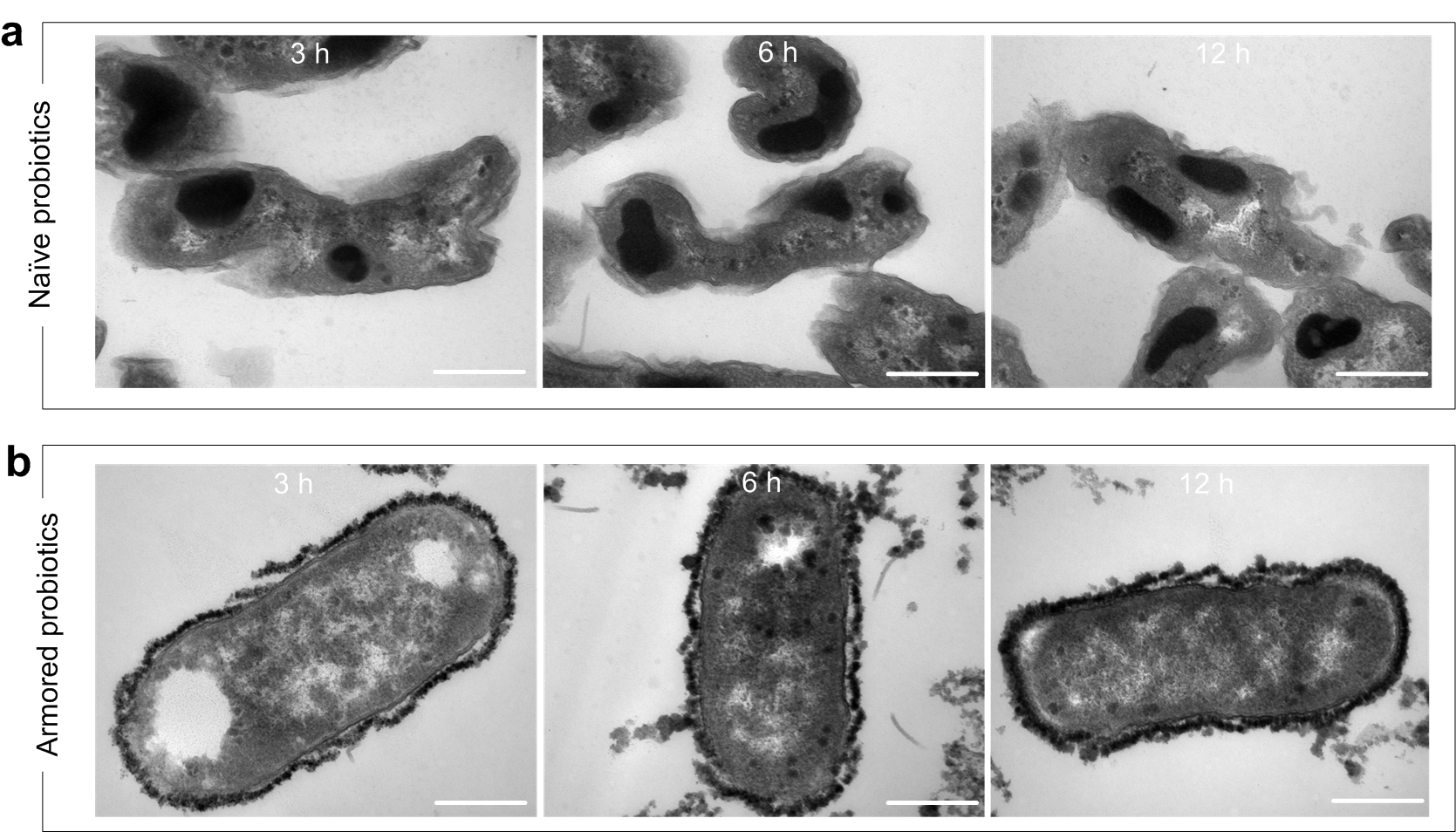


**Figure S15.** The cross-sectional TEM images of (**a**) naïve and (**b**) armored probiotics after treatment of simulated intestinal fluid with levofloxacin for different times. The nanoarmors kept intact due to the significantly slow division rate of bacteria in the simulated intestinal fluid without culture medium. Scale bar, 200 nm.


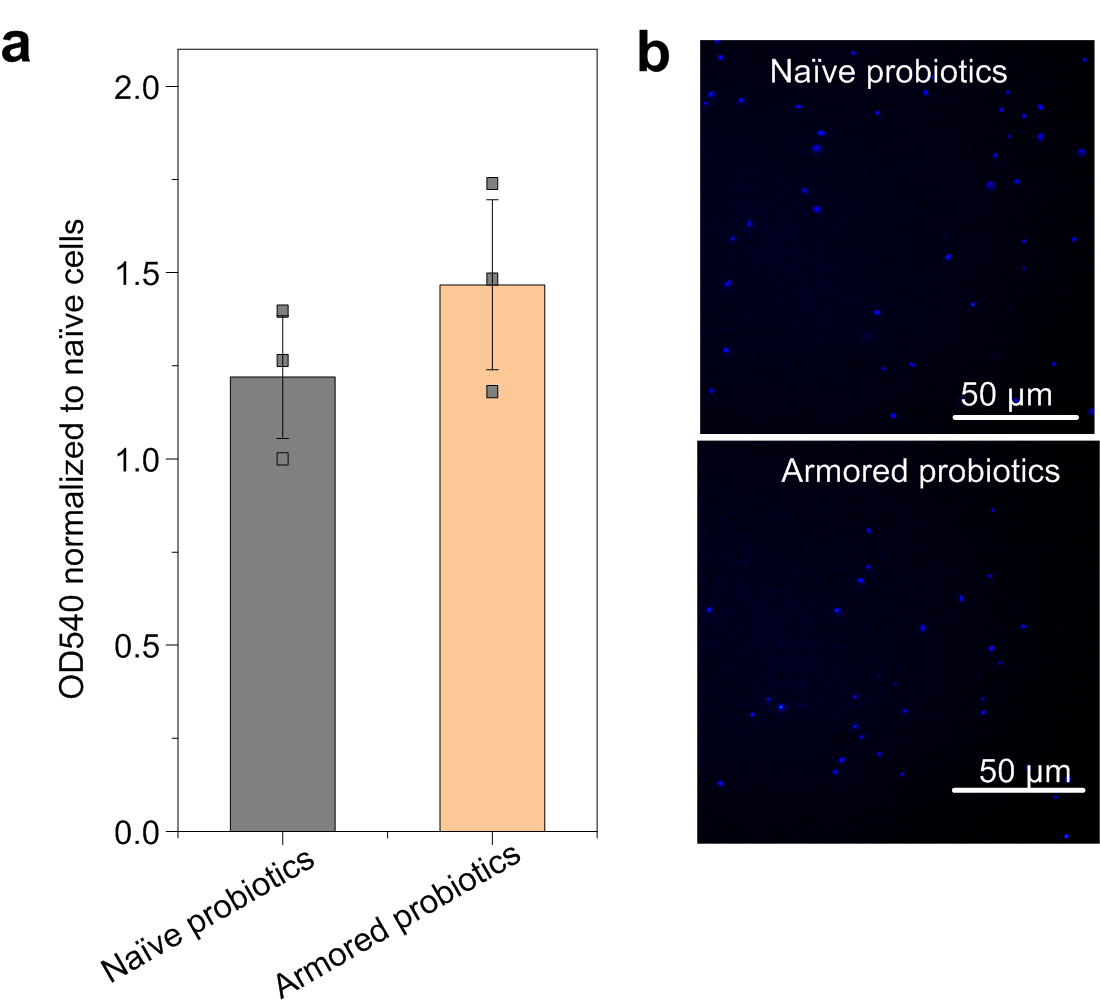


**Figure S16.** (**a**) The adhesion ability of naïve /armored probiotics to intestinal mucus of rats and (**b**) corresponding fluorescent photos. The armored probiotics were able to adhere to intestinal mucus. Therefore, nanoarmor had the potential to promote the colonization of armored probiotics in the intestinal tract. Variation is represented by the standard deviation of three independent replicates in all graphs. The graphs represent mean values ± SEM.


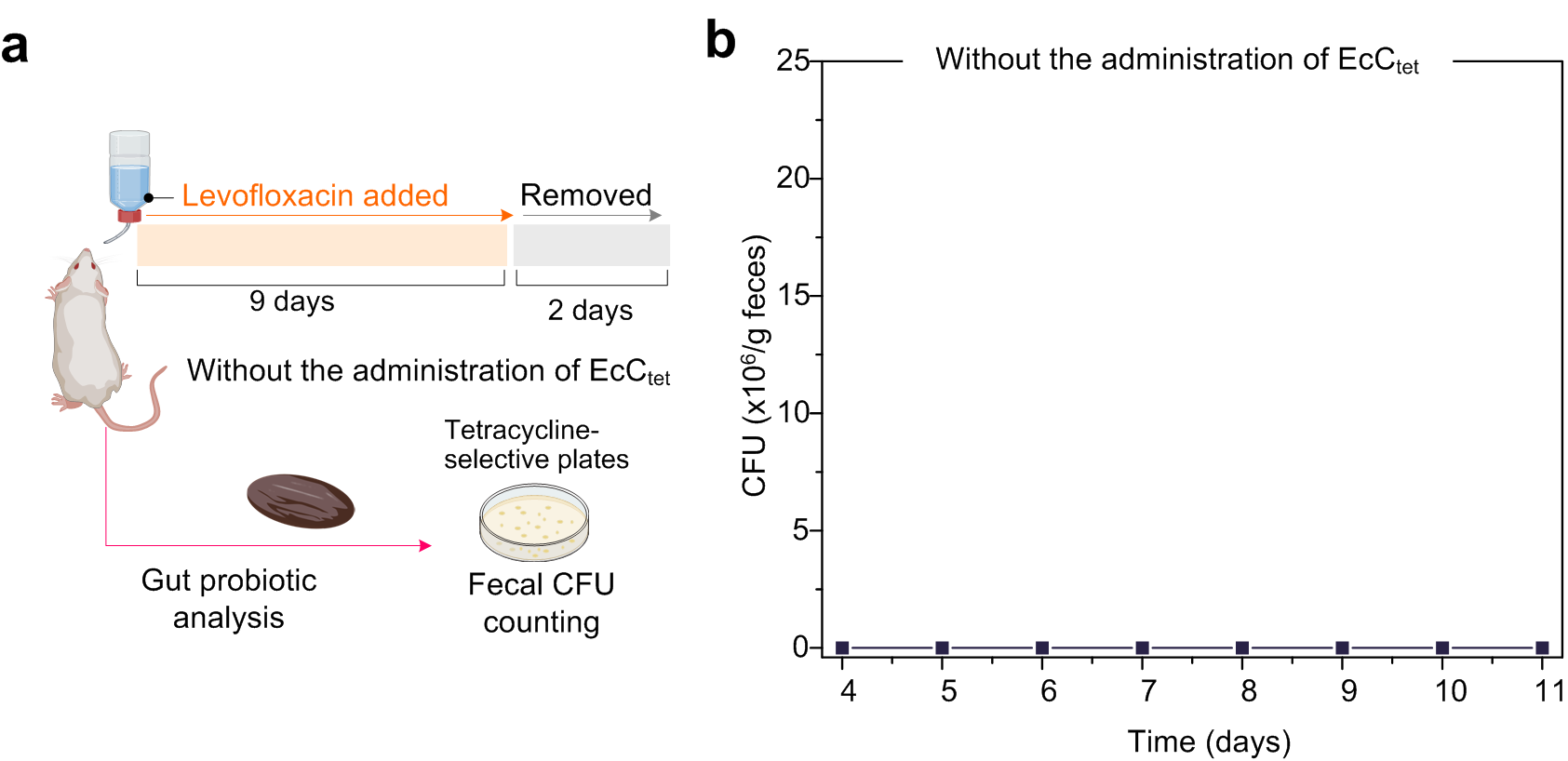


**Figure S17.** (**a**) Schematic representation of animal experiment design. (**b**) Fecal CFU counts of bacteria in rats which could grow on the tetracycline-selective plates. If the rats received levofloxacin without the administration of EcC_tet_, no colonies can be observed on the tetracycline-selective plates throughout the experiments. Variation is represented by the standard deviation of three independent replicates in all graphs.


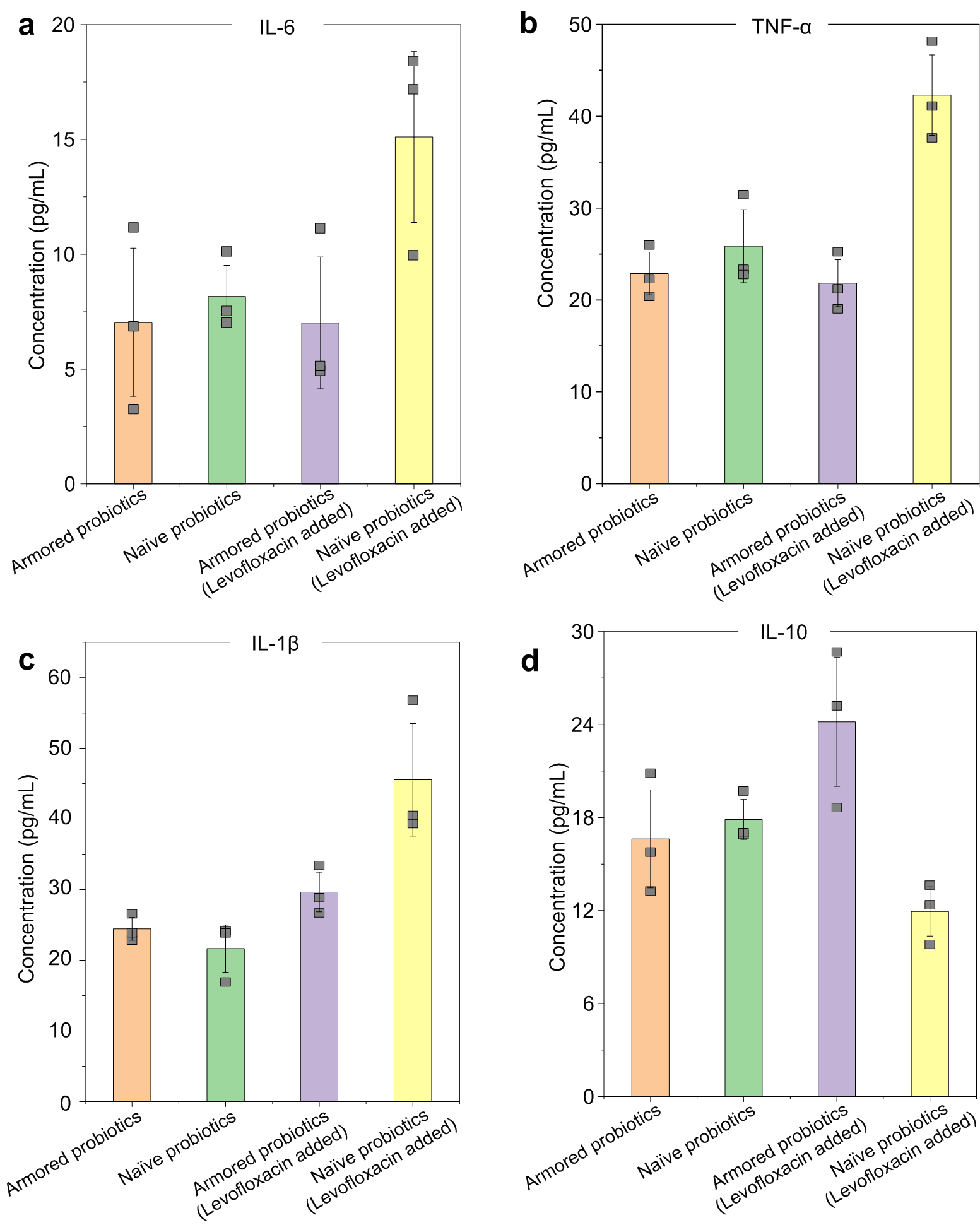


**Figure S18.** (**a**) Interleukin-6 (IL-6), (**b**) tumor necrosis factor-α (TNF-α), (**c**) interleukin-1β (IL-1β), and (**d**) interleukin-10 (IL-10) levels in serum of rats on day 11 measured by ELISA. In contrast to AAD rats (rats with the administration of levofloxacin) treated with naïve EcC_tet_, treating rats with armored EcC_tet_ reduced the levels of proinflammatory cytokines in serum, including IL-6, IL-1β and TNF-α. Treating with armored EcC_tet_ also promoted the expression of anti-inflammatory cytokine IL-10 in the serum. Rats without the treatment of levofloxacin did not show inflammatory responses. Variation is represented by the standard deviation of three independent replicates in all graphs. The graphs represent mean values ± SEM.


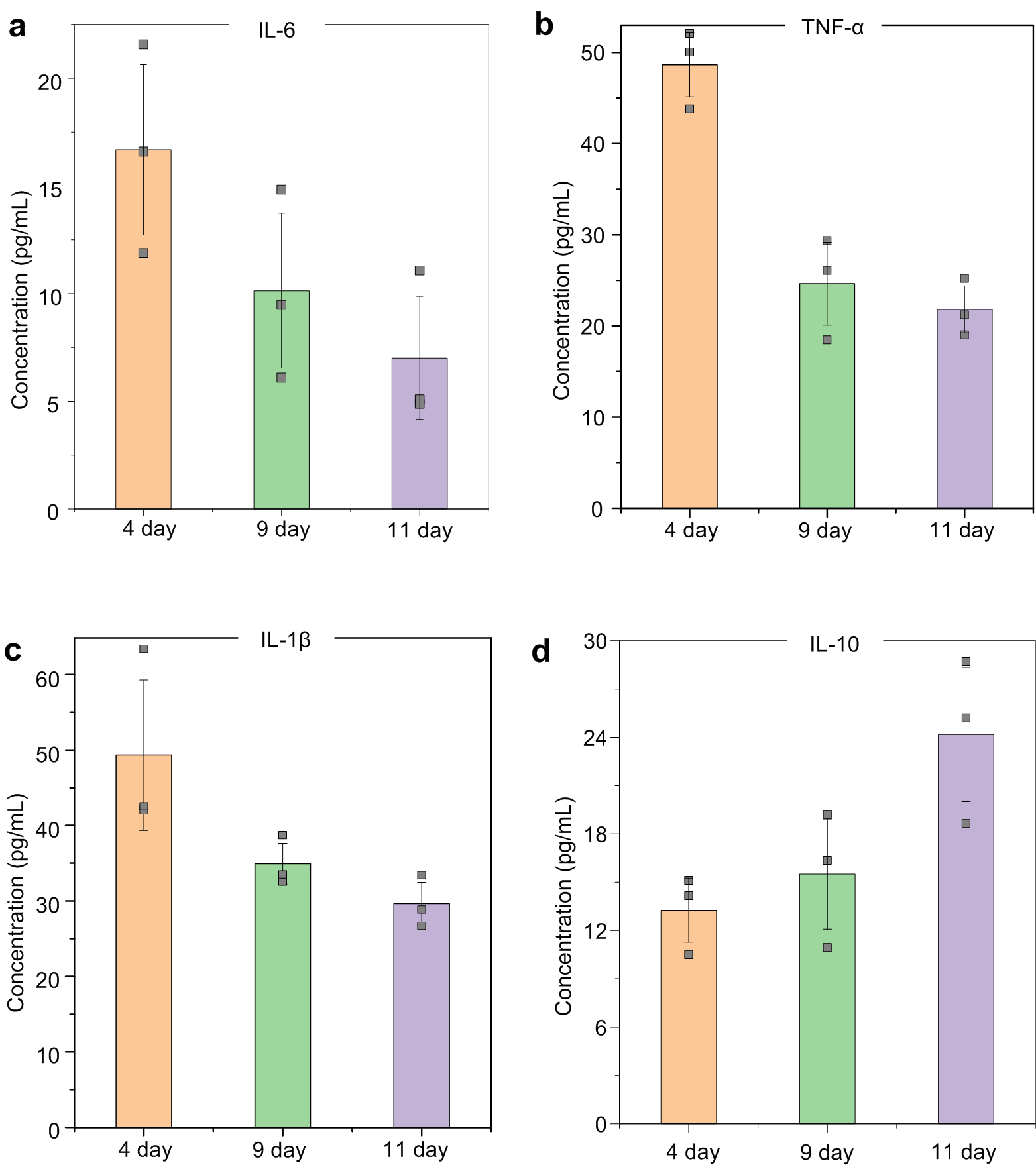


**Figure S19.** (**a**) Interleukin-6 (IL-6), (**b**) tumor necrosis factor-α (TNF-α), (**c**) interleukin-1β (IL-1β), and (**d**) interleukin-10 (IL-10) levels in serum of AAD rats with the administration of armored EcC_tet_ on days 4, 9, and 11 measured by ELISA. After treatment with armored EcC_tet_, rats showed a decrease in the expression of proinflammatory cytokines (IL-6, IL-1β, TNF-α) and an increase in the expression of anti-inflammatory cytokines (IL-10) in the serum at days 9, 11. Variation is represented by the standard deviation of three independent replicates in all graphs. The graphs represent mean values ± SEM.


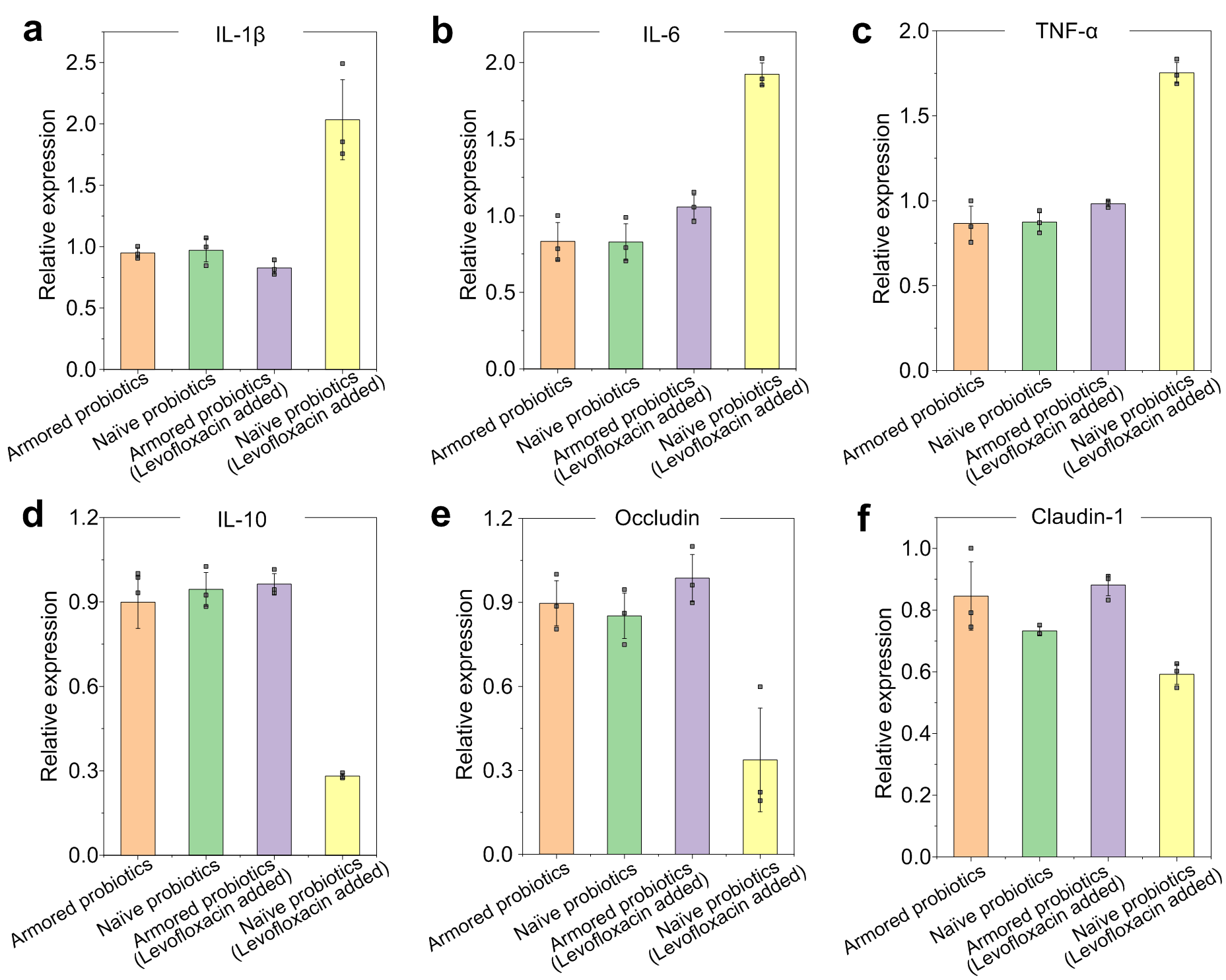


**Figure S20.** RT-qPCR analyses of (**a**) IL-1β, (**b**) IL-6, (**c**) TNF-α, (**d**) IL-10, (**e**) Occludin, and (**f**) Claudin-1 mRNA levels on day 11. In contrast to AAD rats treated with naïve EcC_tet_, administration of armored EcC_tet_ downregulated the genes of pro-inflammatory colonic cytokines in the GI tract, including IL-6, IL-1β and TNF-α. Treating with armored EcC_tet_ also upregulated the gene of anti-inflammatory cytokine (IL-10) and tight junction proteins (Occludin, Claudin-1). Rats without the treatment of levofloxacin did not show inflammatory responses. Variation is represented by the standard deviation of three independent replicates in all graphs. The graphs represent mean values ± SEM.


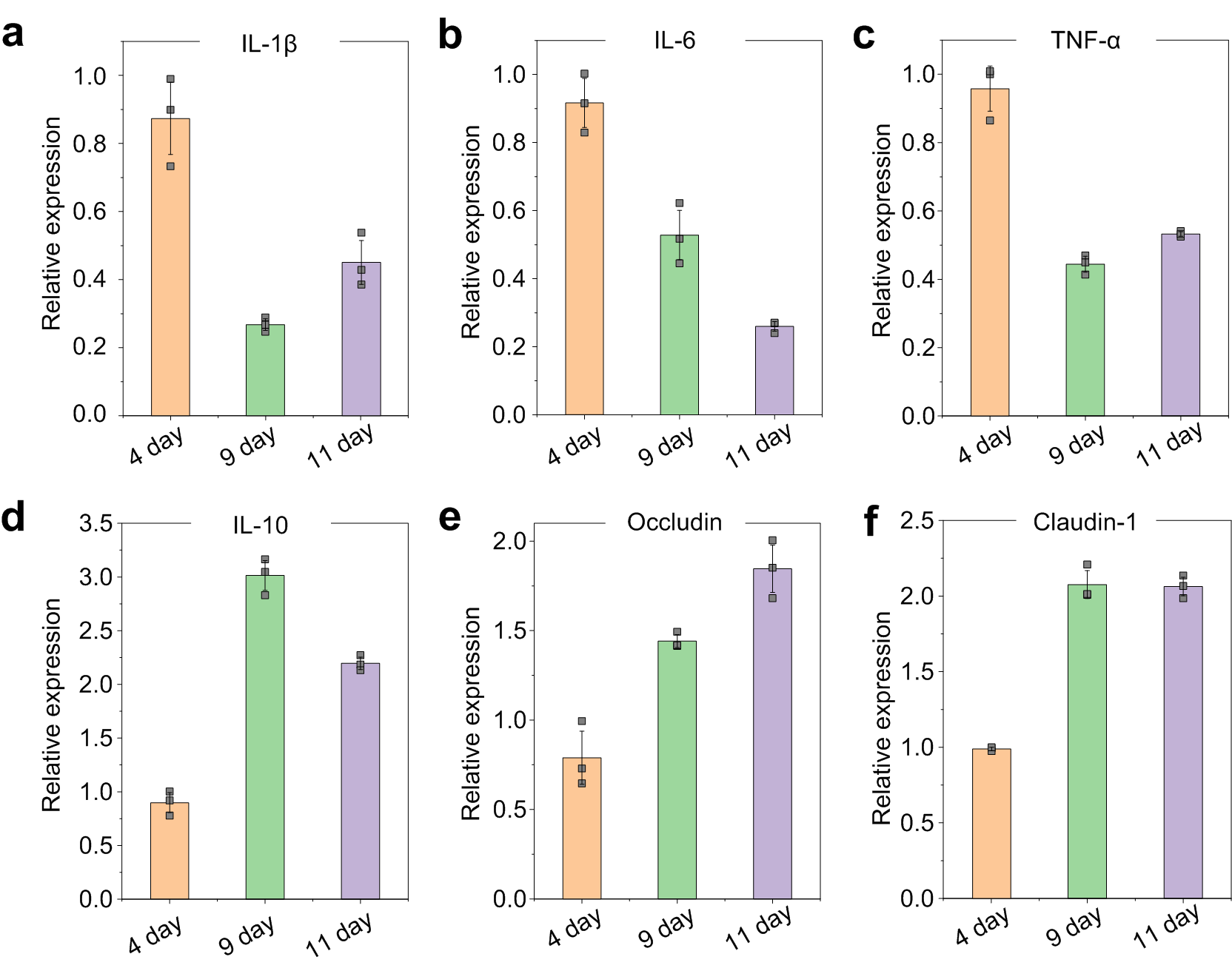


**Figure S21.** RT-qPCR analyses of (**a**) IL-1β, (**b**) IL-6, (**c**) TNF-α, (**d**) IL-10, (**e**) Occludin, and (**f**) Claudin-1 mRNA levels of AAD rats with the administration of armored EcC_tet_ on days 4, 9, and 11. After treatment with armored EcC_tet_, rats downregulated the genes of pro-inflammatory colonic cytokines (IL-6, IL-1β, and TNF-α), upregulated anti-inflammatory cytokine (IL-10), and tight junction proteins (Occludin, Claudin-1) at days 9 and 11. Variation is represented by the standard deviation of three independent replicates in all graphs. The graphs represent mean values ± SEM.


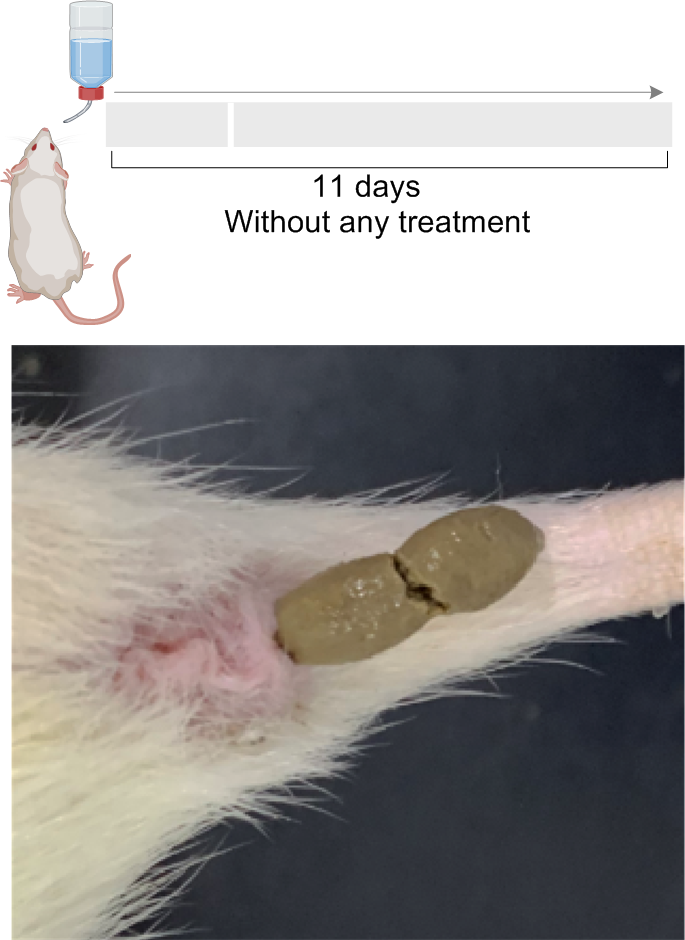


**Figure S22.** The representative images of the corresponding fecal samples for the healthy rats without any treatment. The fecal particle was normal in shape, and was dark-brown in color.


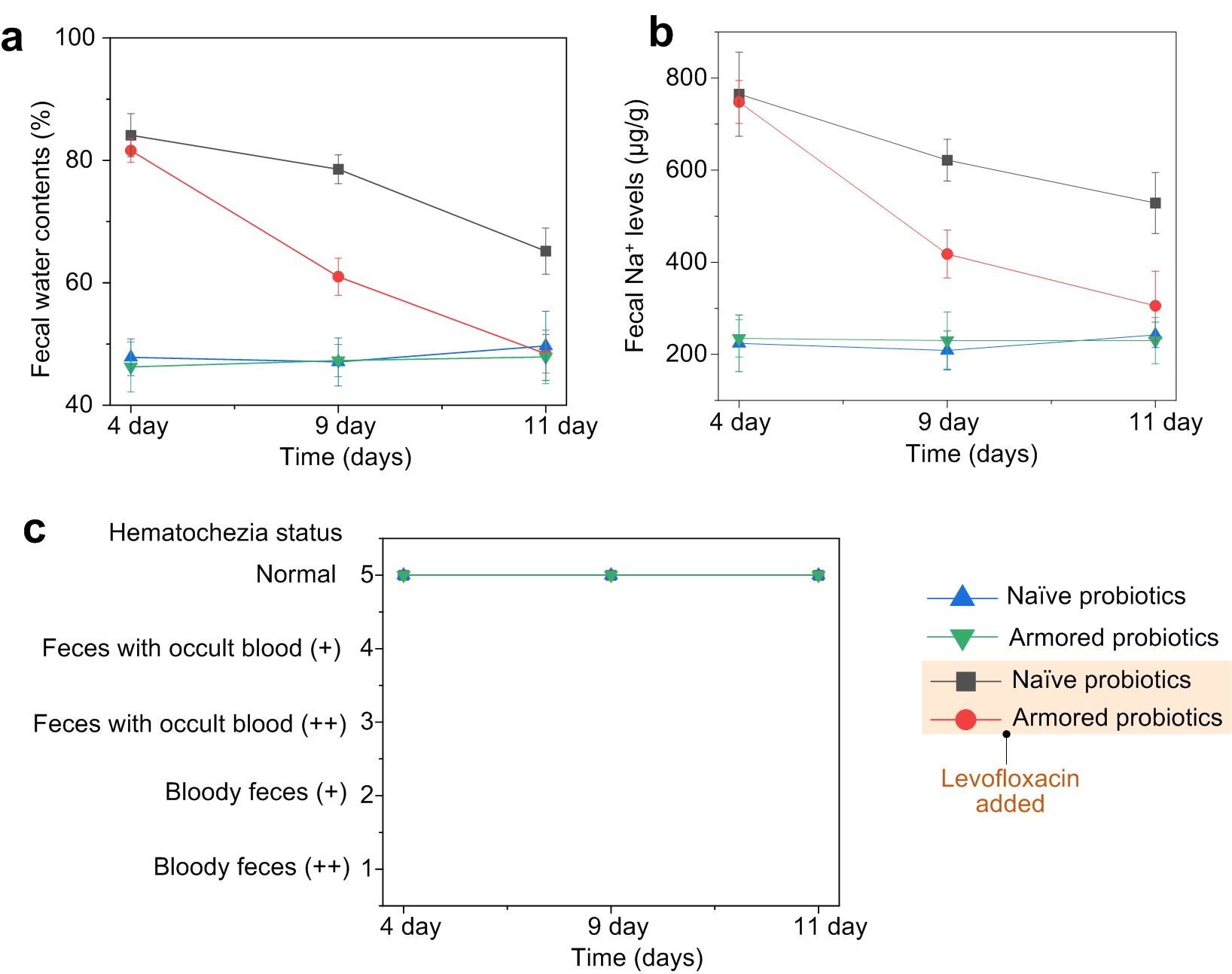


**Figure S23.** (**a**) Fecal water contents of rats measured on days 4, 9, and 11. (**b**) Fecal Na^+^ levels of rats measured on days 4, 9, and 11. (**c**) Fecal hematochezia status of rats measured on days 4, 9, and 11. Variation is represented by the standard deviation of three independent replicates in all graphs. The graphs represent mean values ± SEM.


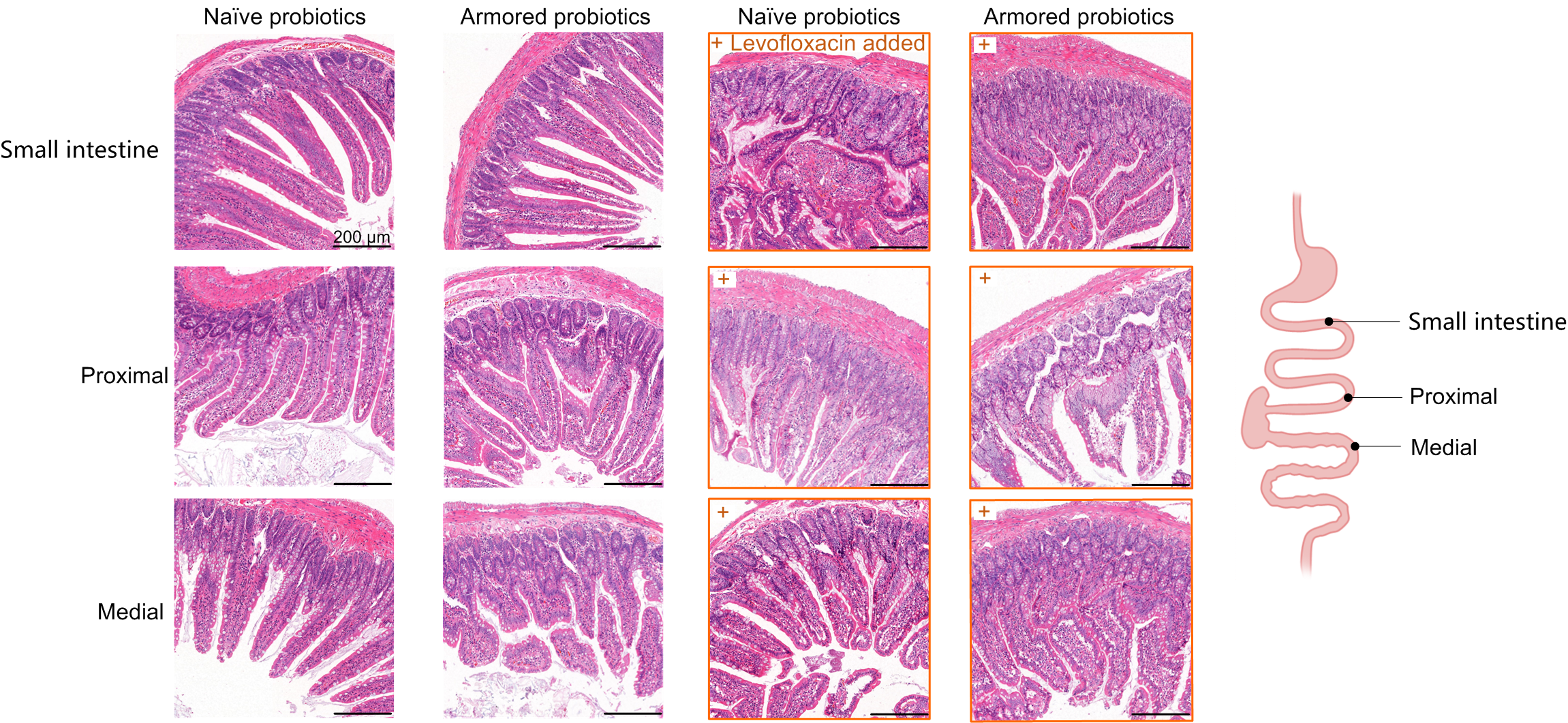


**Figure S24.** Representative histological sections obtained from the GI tissues of each cohort, visualized with H&E stain. No detrimental physiological effects were observed in any of the animals. Scale bars, 200 μm.


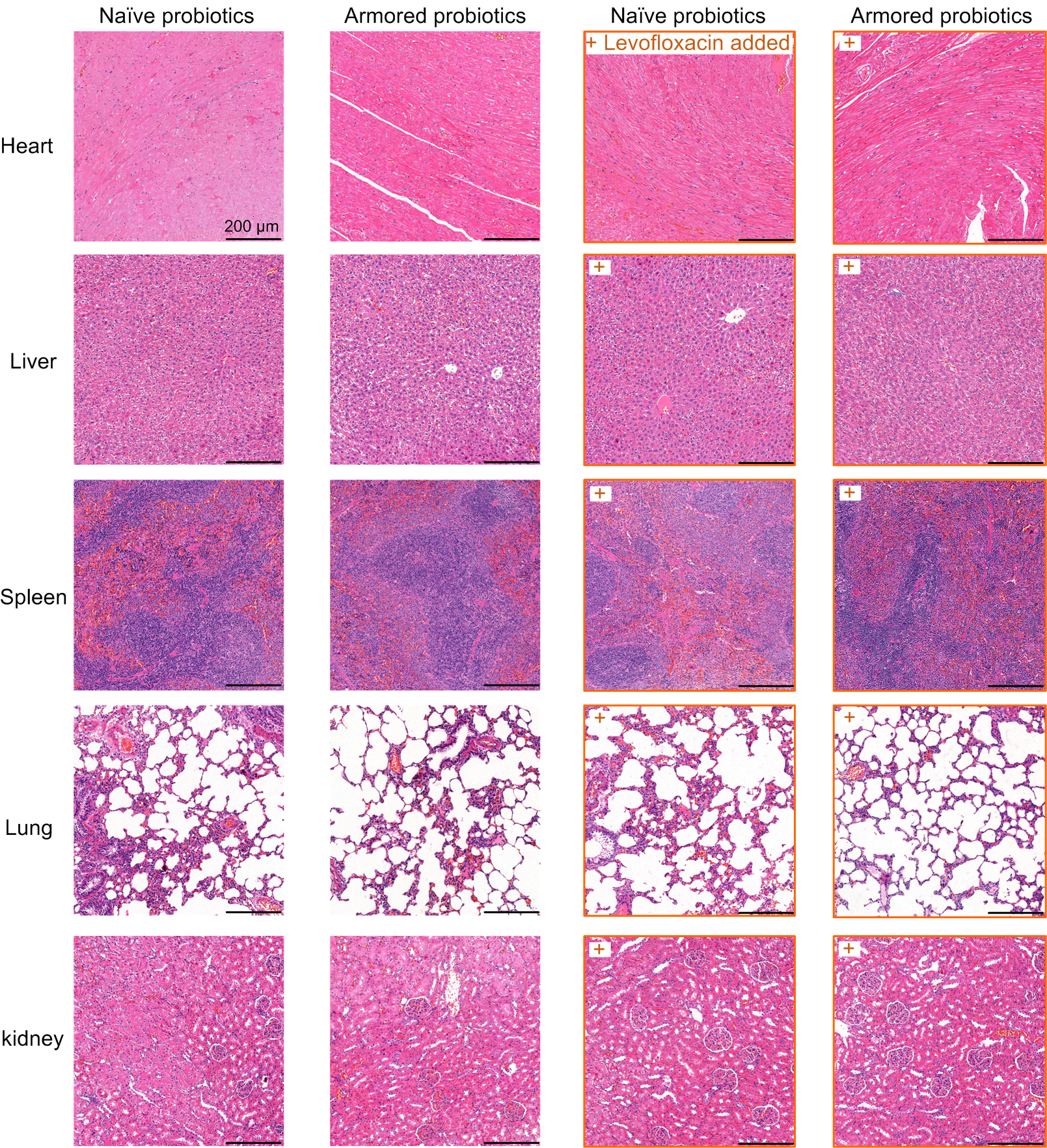


**Figure S25.** Representative histological sections obtained from the tissues of each cohort, visualized with H&E stain. No detrimental physiological effects were observed in any of the animals. Scale bars, 200 μm.


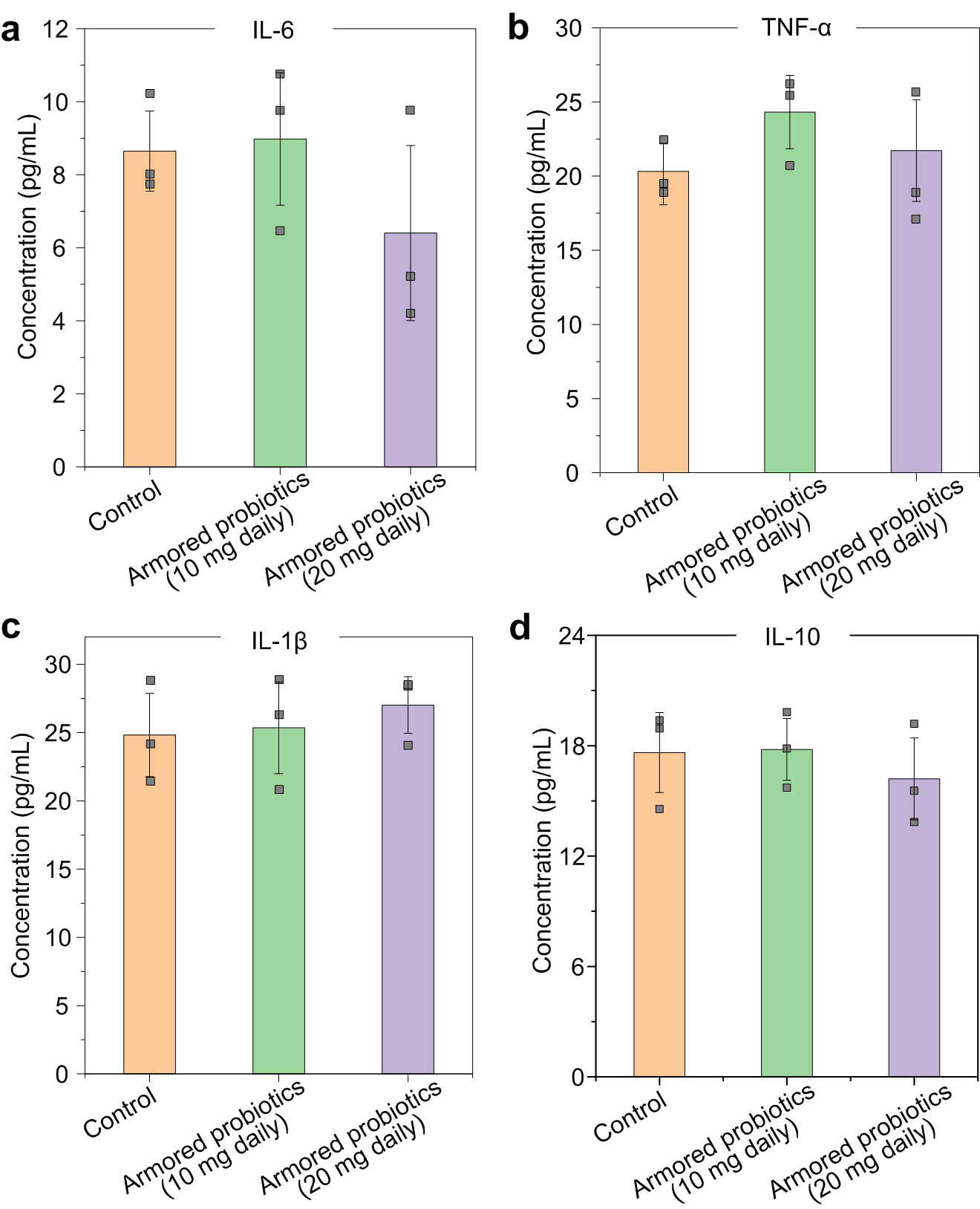


**Figure S26.** (**a**) IL-6, (**b**) TNF-α, (**c**) IL-1β, and (**d**) IL-10 levels in the serum of rats in the daily administration of armored EcC_tet_ for 6 days. The results showed that the expression of pro-inflammatory and anti-inflammatory factors (IL-6, IL-1β, TNF-α, IL-10) in the serum of each rat was normal. There was no significant difference in all indicators between the rats who received armored probiotics and the rats as controls. Variation is represented by the standard deviation of three independent replicates in all graphs. The graphs represent mean values ± SEM.


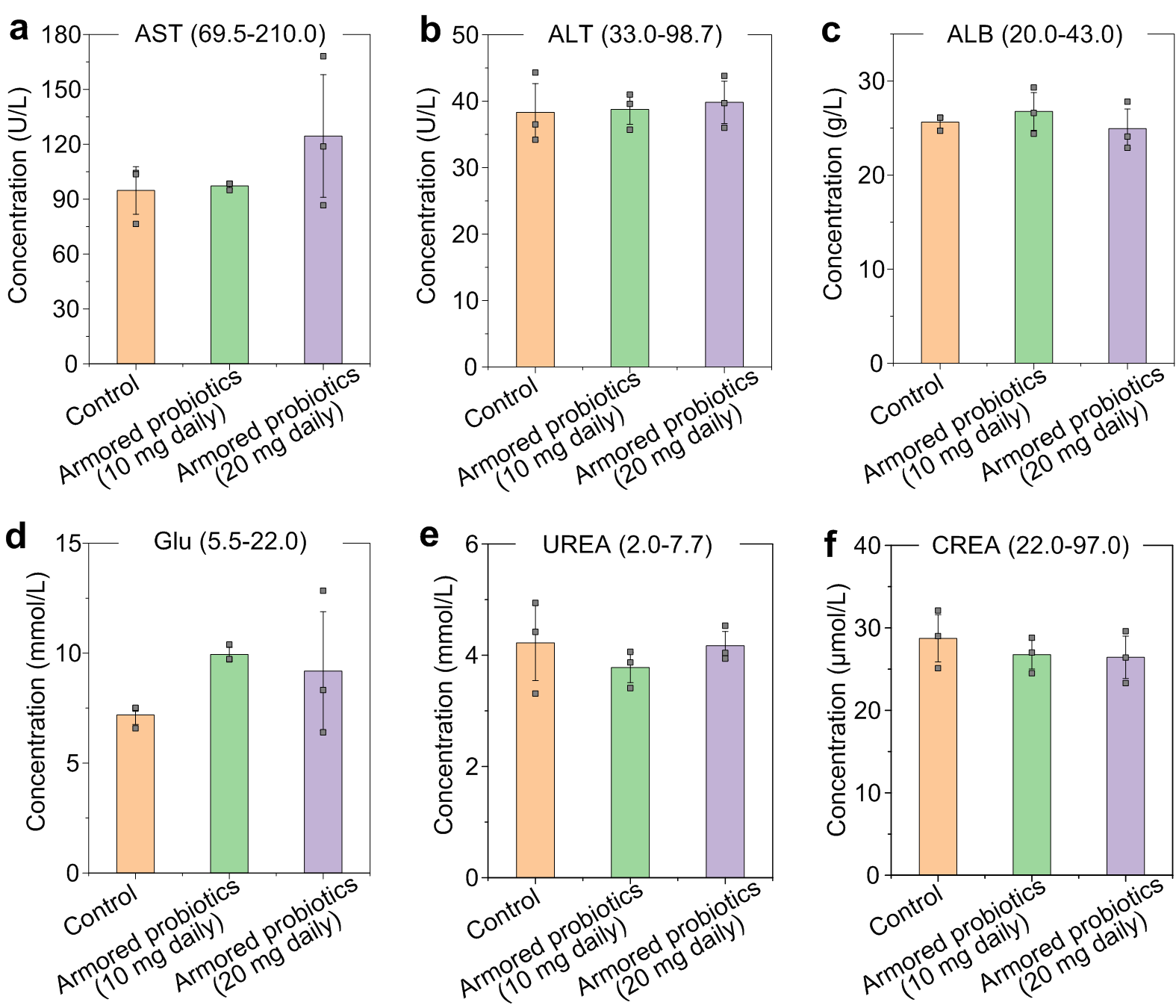


**Figure S27.** Biochemical analysis of rats with different administration of armored EcC_tet_: (**a**) aspartate aminotransferase (AST), (**b**) alanine aminotransferase (ALT), (**c**) albumin (ALB), (**d**) glucose (Glu), (**e**) urea, (**f**) creatinine (CREA). There was no significant difference in all indicators between the rats who received armored probiotics and the rats as controls. Variation is represented by the standard deviation of three independent replicates in all graphs. The graphs represent mean values ± SEM.


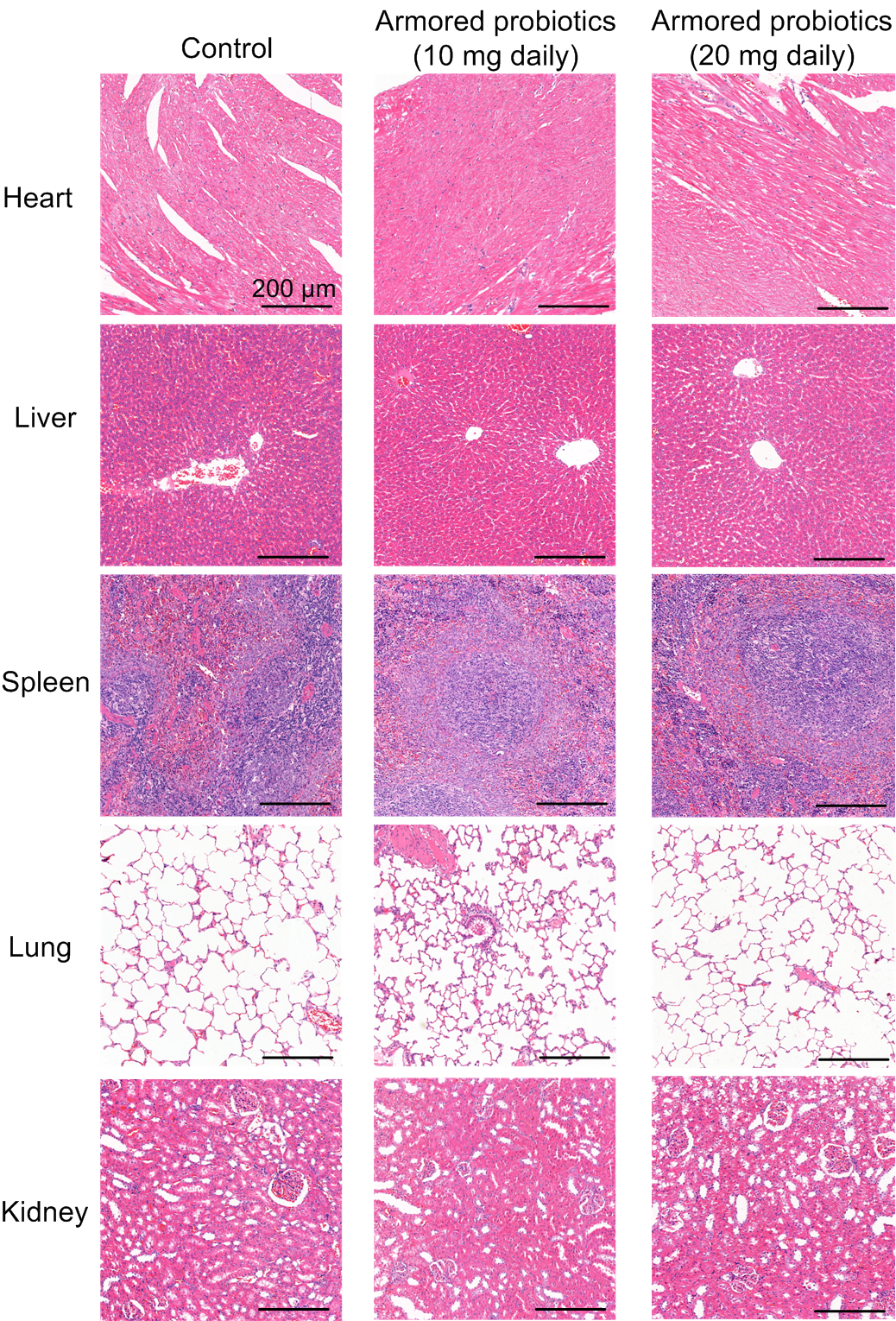


**Figure S28.** Representative histological sections obtained from the tissues of each cohort, visualized with H&E stain. No detrimental physiological effects were observed in any of the animals. Scale bars, 200 μm.


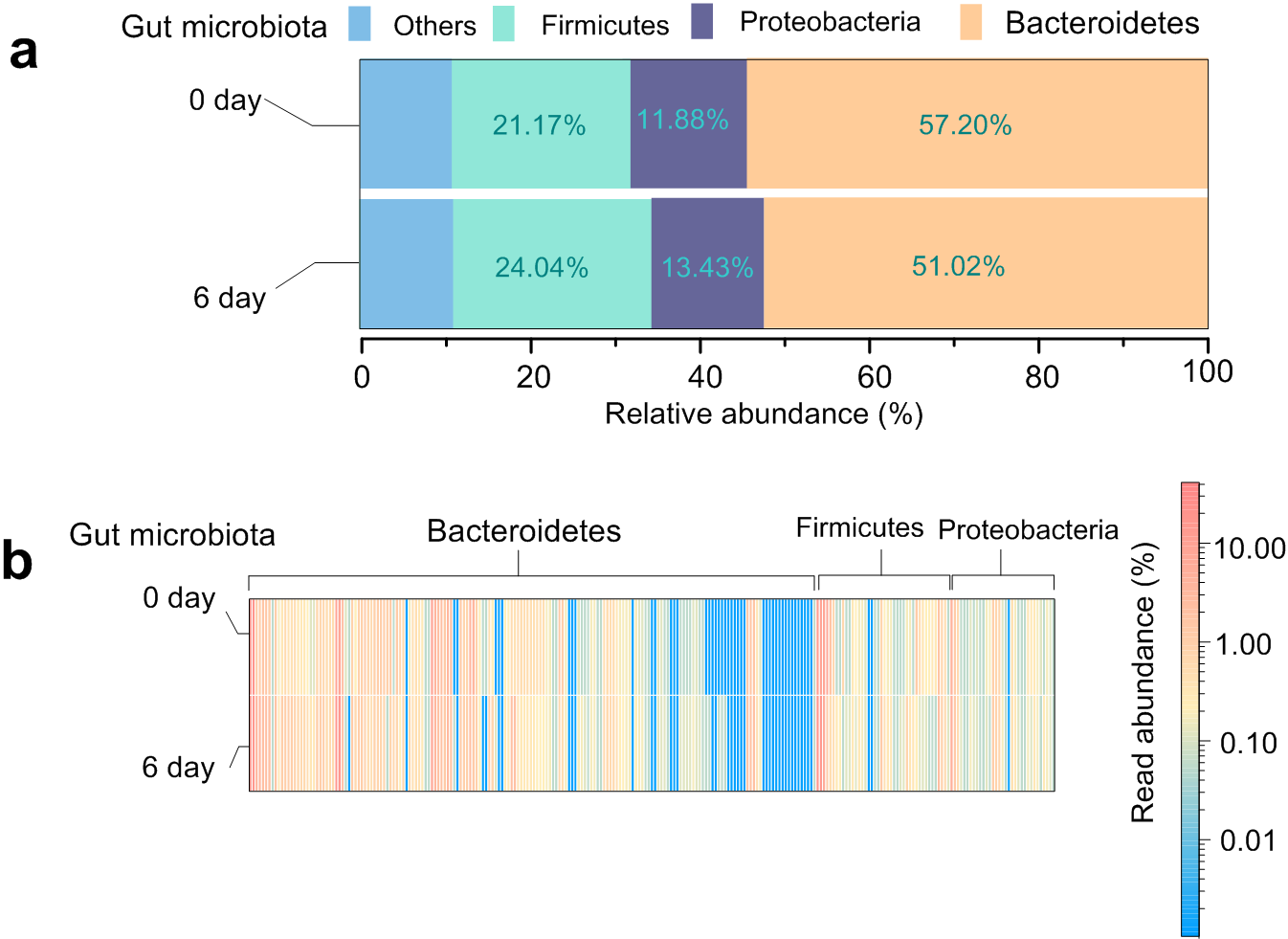


**Figure S29.** (**a**) Taxa summary map of different phyla in gut microbiota before/after the administration of armored probiotics. (**b**) Heat map of metagenomic sequencing results of different strains of gut microbiota before/after the administration of armored probiotics.

**Supplementary References**

1. Guo, J. *et al.* Modular assembly of superstructures from polyphenol-functionalized building blocks. *Nat. Nanotech.* **11**, 1105-1111 (2016).

2. Guo, J. *et al.* Engineering multifunctional capsules through the assembly of metal–phenolic networks. *Angew. Chem. Int. Ed.* **53**, 5546-5551 (2014).

3. Zhao, Z. *et al.* Engineering of living cells with polyphenol‐functionalized biologically active nanocomplexes. *Adv. Mater.* **32**, 2003492 (2020).
